# The evolution of the platyrrhine talus: A comparative analysis of the phenetic affinities of the Miocene platyrrhines with their modern relatives

**DOI:** 10.1016/j.jhevol.2017.07.015

**Published:** 2017-10

**Authors:** Thomas A. Püschel, Justin T. Gladman, René Bobe, William I. Sellers

**Affiliations:** a School of Earth and Environmental Sciences, University of Manchester, M13 9PL, United Kingdom; b Department of Anthropology, The Graduate Center, CUNY, New York, NY, USA; c NYCEP, New York Consortium in Evolutionary Primatology, New York, NY, USA; d Departamento de Antropología, Universidad de Chile, Santiago, Chile; e Institute of Cognitive and Evolutionary Anthropology, School of Anthropology, University of Oxford, United Kingdom

**Keywords:** New World monkeys, Talar morphology, Geometric morphometrics, Locomotor mode percentages, Phylogenetic comparative methods, Body mass prediction

## Abstract

Platyrrhines are a diverse group of primates that presently occupy a broad range of tropical-equatorial environments in the Americas. However, most of the fossil platyrrhine species of the early Miocene have been found at middle and high latitudes. Although the fossil record of New World monkeys has improved considerably over the past several years, it is still difficult to trace the origin of major modern clades. One of the most commonly preserved anatomical structures of early platyrrhines is the talus. This work provides an analysis of the phenetic affinities of extant platyrrhine tali and their Miocene counterparts through geometric morphometrics and a series of phylogenetic comparative analyses. Geometric morphometrics was used to quantify talar shape affinities, while locomotor mode percentages (LMPs) were used to test if talar shape is associated with locomotion. Comparative analyses were used to test if there was convergence in talar morphology, as well as different models that could explain the evolution of talar shape and size in platyrrhines. Body mass predictions for the fossil sample were also computed using the available articular surfaces. The results showed that most analyzed fossils exhibit a generalized morphology that is similar to some ‘generalist’ modern species. It was found that talar shape covaries with LMPs, thus allowing the inference of locomotion from talar morphology. The results further suggest that talar shape diversification can be explained by invoking a model of shifts in adaptive peak to three optima representing a phylogenetic hypothesis in which each platyrrhine family occupied a separate adaptive peak. The analyses indicate that platyrrhine talar centroid size diversification was characterized by an early differentiation related to a multidimensional niche model. Finally, the ancestral platyrrhine condition was reconstructed as a medium-sized, generalized, arboreal, quadruped.

## 1 Introduction

Modern New World monkeys (NWM) occupy a diverse array of habitats, ranging from the Amazonian Basin, the semi-deciduous Atlantic Forest, to the fringes of great forests such as in the Venezuelan plains (Rylands and Mittermeier, 2009, Fleagle, 2013). The occupation of these diverse environments has been accompanied by distinct behavioral, morphological and ecological adaptations, which are broadly correlated with specific phylogenetic groups (Ford and Davis, 1992, Rosenberger, 1992, Fleagle and Reed, 1996, Fleagle et al., 1999, Rosenberger, 2002, Youlatos, 2004, Rosenberger et al., 2009). Whilst the modern day success of the group is clear, the evolutionary history of these lineages is still highly debated (Youlatos and Meldrum, 2011). Currently one of the main difficulties in platyrrhine paleontology is the scarcity of data available from the Eocene and Oligocene, because most platyrrhine fossils have been dated to the Miocene or the Pleistocene of South America and the Caribbean (Rímoli, 1977, MacPhee and Woods, 1982, MacPhee et al., 2003, Kay and Cozzuol, 2006, Tejedor et al., 2006, Fleagle et al., 2012, Perkins et al., 2012), although there are notable exceptions from Bolivia and Peru (Hoffstetter, 1969, Rosenberger, 1981, Wolff, 1984, Rosenberger et al., 1991, Takai and Anaya, 1996, Takai et al., 2000, Kay et al., 2002, Bond et al., 2015). Most of these fossils are composed of fragmentary dental remains, with several species, such as *Branisella boliviana* (Hoffstetter, 1969), *Mohanimico hershkovitzi* (Luchterhand et al., 1986), *Szalatavus attricuspis* (Rosenberger et al., 1991)*, Solimoea acrensis* (Kay and Cozzuol, 2006), *Insulacebus toussainatiana* (Cooke et al., 2011), *Perupithecus ucayaliensis* (Bond et al., 2015), *Panamacebus transitus* (Bloch et al., 2016) and *Canaanimico amazonensis* (Marivaux et al., 2016a), being classified based on limited dental traits.

Interestingly, most of the fossil platyrrhine species of the early Miocene have been found at middle and high latitudes (i.e., central Chile and Patagonia), which are areas that are nowadays uninhabited by non-human primates (Bordas, 1942, Fleagle and Bown, 1983, Fleagle et al., 1987, Fleagle and Kay, 1989, Fleagle, 1990, Meldrum, 1990, Flynn et al., 1995, Tejedor, 2002, Tejedor, 2003, Tejedor, 2005a, Tejedor, 2005b), as well as one from a tropical-equatorial area (i.e., Peruvian Amazonia) (Marivaux et al., 2012) and one from Panama (Bloch et al., 2016). Even though the NWM fossil record has improved considerably over the past several years (Tejedor, 2008, Bond et al., 2015, Kay, 2015a, Bloch et al., 2016, Marivaux et al., 2016a, Marivaux et al., 2016b), it is still difficult to trace the origin of major modern clades (i.e., Atelidae, Pitheciidae and Cebidae), especially considering that some of the earliest fossil taxa may fall outside the crown radiation (Kay et al., 2008, Hodgson et al., 2009, Kay and Fleagle, 2010, Youlatos and Meldrum, 2011; but for a different opinion see Schrago, 2007, Rosenberger, 2010). There are two diverging positions regarding the relationship between the early platyrrhine fossils and the modern species that have been proposed: the long lineage hypothesis (LLH) and the stem platyrrhine hypothesis (SPH) (Kay et al., 2008). The LLH states that modern platyrrhines are defined by a number of long-lived clades and that most of the known fossil taxa belong to these lineages (Rosenberger et al., 2009). This position is supported by some divergence date estimates based on molecular clock data (Schneider et al., 2001, Opazo et al., 2006, Schrago, 2007). The SPH proposes that most of the early Patagonian fossil taxa are not ancestral to the modern clades (Kay et al., 2008, Kay and Fleagle, 2010). Instead they represent a sister group of all living platyrrhines that occupied niches analogous to those filled by modern NWM (Kay et al., 2008, Hodgson et al., 2009, Kay and Fleagle, 2010). Kay and Fleagle (2010) indicate that dissimilar methods can produce varying results starting from the same data and that alternate divergence times lend support to the SPH. Nonetheless, it is important to consider that a phylogenetic meta-analysis carried out by Perez and Rosenberger (2014) comparing the topologies of the 31 major neontological phylogenies concluded that major disparities are rather common among the hypotheses concerning higher level relationships of platyrrhines (e.g., the position of *Aotus*). Additionally, they also found that the correspondence among phylogenetic trees seems to depend on the type of dataset analyzed (i.e., nuclear DNA, mtDNA, Alu sequences, morphology or mixed data), which implies that the biological characteristics emphasized in different datasets intrinsically influence the likelihood of producing similar reconstructions (Perez and Rosenberger, 2014).

One of the most commonly preserved anatomical elements in the platyrrhine fossil record is the talus (Tejedor, 2008). Many Argentinian platyrrhine taxa exhibit at least one preserved talus (i.e., *Carlocebus carmenensis, Soriacebus ameghinorum, Dolichocebus gaimanensis, Proteropithecia neuquenensis*), while in Chile (Río Cisnes) and Peru (Madre de Dios) the post-cranial fossil record is represented by tali (Bordas, 1942, Fleagle and Bown, 1983, Fleagle et al., 1987, Fleagle and Kay, 1989, Fleagle, 1990, Meldrum, 1990, Flynn et al., 1995, Tejedor, 2002, Tejedor, 2003, Tejedor, 2005a, Tejedor, 2005b, Marivaux et al., 2012). Many of the Colombian fossils from La Venta also have preserved tali (i.e., *Neosamiri fieldsi*, *Aotus dindensis, Cebupithecia sarmientoi*) and the Miocene Caribbean fossil of *Paralouatta marianae* is represented only by one talus (MacPhee et al., 2003). Furthermore, the talus is important because it has been suggested that its morphology could reflect postural adaptations, based on its central position in the foot as well as its functional relationship with other foot bones (Lisowski et al., 1974, Boyer et al., 2010, Boyer et al., 2015, Yapuncich and Boyer, 2014, Yapuncich et al., 2015). The talus is the principal mechanical link between the leg and the foot, hence it is responsible for transmitting forces derived from an animal's body mass, as well as allowing mobility and providing stability during most postural and locomotor behaviors (Boyer et al., 2015). Consequently, it has been argued that the talus is a useful element for both functional and phylogenetic analyses based on its high prevalence and good preservation in the fossil record, and also because its intricate morphology coupled with a relatively straightforward functional role in the ankle joint allow postural and locomotor inferences (Gebo, 1986, Gebo, 1988, Gebo, 2011, Boyer and Seiffert, 2013). Even though some platyrrhine fossil tali have been analyzed using linear morphometrics (Meldrum, 1990), there is an absence of current morphometric and comparative analyses that could provide important information regarding the evolution of this anatomical structure.

In this study we analyze Miocene fossil platyrrhine talar shape and size in the context of a broad comparative sample representing all extant platyrrhine families. Modern NWM are represented by three families that are well-defined based on congruent morphological and molecular data (Aristide et al., 2015, Kay, 2015b), except for the still debated position of *Aotus*, which has been classified either as a member of the cebines, as a sister group of the callitrichines or as a pithecid (Kay, 1990, Rosenberger et al., 1990, Rosenberger, 2002, Wildman et al., 2009). These clades show remarkable adaptions to different environments, occupying very distinct habitats and climates. Consequently their ecomorphological adaptations and body sizes are variable, ranging in the modern platyrrhine clade from 100 g to more than 10,000 g (Ford and Davis, 1992). Thus, this research has four objectives. First, to examine morphological affinities, and identify the phenetic affinities between fossil and living NWM tali. Second, to analyze locomotor mode percentages to understand the relationship between locomotion and talar shape and reconstruct the ancestral locomotor condition of the NWM. Third, to undertake evolutionary modeling to test if there is morphological convergence among NWMs and model the possible evolutionary processes explaining observed diversity in talar shape and size. Fourth, to predict body mass for the fossil sample.

## 2 Material and methods

### 2.1 Sample

The comparative sample included platyrrhines from nearly every extant genus in order to capture the full morphological diversity of the extant crown group (*n* = 203; 40 species; Table 1). The fossil sample included most of the available Miocene platyrrhine tali (*n* = 15; eight species plus two specimens that have not been taxonomically assigned; Table 2). A total of 34 three-dimensional (3D) tali scans were downloaded from Morphosource (http://morphosource.org/) – an online repository of 3D scan data (Copes et al., 2016) – as ply surface models, while the rest were scanned for this study (details of the sample are provided in the Supplementary Online Material [SOM] S1).

**Table 1:** Extant sample.

Species	*n*	Postural behavior
*Alouatta caraya*	16	Clamber/suspensory
*Alouatta seniculus*	15	Clamber/suspensory
*Aotus azarae*	19	Arboreal quadrupedalism
*Aotus infulatus*	1	Arboreal quadrupedalism
*Aotus nancymaae*	2	Arboreal quadrupedalism
*Aotus trivirgatus*	3	Arboreal quadrupedalism
*Ateles belzebul*	6	Clamber/suspensory
*Ateles fusciceps*	3	Clamber/suspensory
*Ateles geoffroyi*	4	Clamber/suspensory
*Ateles marginatus*	2	Clamber/suspensory
*Cacajao calvus*	8	Arboreal quadrupedalism
*Callicebus cupreus*	3	Arboreal quadrupedalism
*Callicebus donacophilus*	5	Arboreal quadrupedalism
*Callicebus moloch*	4	Arboreal quadrupedalism
*Callicebus personatus*	1	Arboreal quadrupedalism
*Callicebus torquatus*	1	Arboreal quadrupedalism
*Callimico goeldii*	7	Leaper/clawed
*Callithrix geoffroyi*	2	Leaper/clawed
*Callithrix jacchus*	8	Leaper/clawed
*Callithrix penicillata*	1	Leaper/clawed
*Cebuella pygmaea*	5	Leaper/clawed
*Cebus albifrons*	10	Arboreal quadrupedalism
*Cebus apella*	14	Arboreal quadrupedalism
*Cebus nigritus*	1	Arboreal quadrupedalism
*Cebus olivaceus*	5	Arboreal quadrupedalism
*Chiropotes satanas*	4	Arboreal quadrupedalism
*Lagothrix lagotricha*	5	Clamber/suspensory
*Leontopithecus rosalia*	5	Leaper/clawed
*Mico argentatus*	1	Leaper/clawed
*Mico humeralifer*	1	Leaper/clawed
*Mico melanurus*	1	Leaper/clawed
*Pithecia monachus*	1	Arboreal quadrupedalism
*Pithecia pithecia*	2	Arboreal quadrupedalism
*Saguinus fuscicollis*	1	Leaper/clawed
*Saguinus leucopus*	1	Leaper/clawed
*Saguinus midas*	6	Leaper/clawed
*Saguinus mystax*	6	Leaper/clawed
*Saguinus oedipus*	1	Leaper/clawed
*Saimiri boliviensis*	16	Arboreal quadrupedalism
*Saimiri sciureus*	6	Arboreal quadrupedalism
Total	203

**Table 2:** Fossil sample.

Fossil	Age (Ma)	Locality	Previous body mass estimates (g)	Accession number
*Dolichocebus gaimanensis*	∼20.0	Sarmiento, Chubut, Argentina	1500	MACN 362
*Carlocebus carmenensis* (*n* = 4)	17.5–16.5	Pinturas, Santa Cruz, Argentina	2500	MACN 271, 304, 368, 396
*Soriacebus ameghinorum*	17.5–16.5	Pinturas, Santa Cruz, Argentina	1800	MACN 397
Madre de Dios	∼18.75–16.5	Atalaya, Cusco, Upper Madre de Dios Basin, Peru	250–500	MUSM 2024
Río Cisnes	16.5	Alto Río Cisnes, Chile	?	SGO.PV 974
*Proteropithecia neuquenensis*	15.8	Collón Curá, Neuquén, Argentina	1500	MLP 91-IX-1-119
*Aotus dindensis*^a^	13.0–13.2	La Venta, Madgalena Valley, Colombia	1000	IGMKU 8802
*Cebupithecia sarmientoi*	13.5–11.8	La Venta, Madgalena Valley, Colombia	1602	UCMP 38762
*Neosaimiri fieldsi* (*n* = 3)^a^	12.0–13.2	La Venta, Madgalena Valley, Colombia	725	IGMKU 89030, 89031, 89199
*Paralouatta marianae*^a^	∼17.5–18.5	Domo de Zaza, Lagunitas Formation, Cuba	?	MNHNCu 76.3059

a Scans obtained from casts.

### 2.2 3D surface rendering

Surface models were imported into Geomagic Studio v. 12 (Geomagic, USA). Using this software, possible errors in the polygon mesh were identified and adjusted to remove localized holes and protruding vertices. When the 3D models where particularly large, they were globally re-meshed to simplify their element geometry.

### 2.3 Morphological affinities

**Figure 1:**
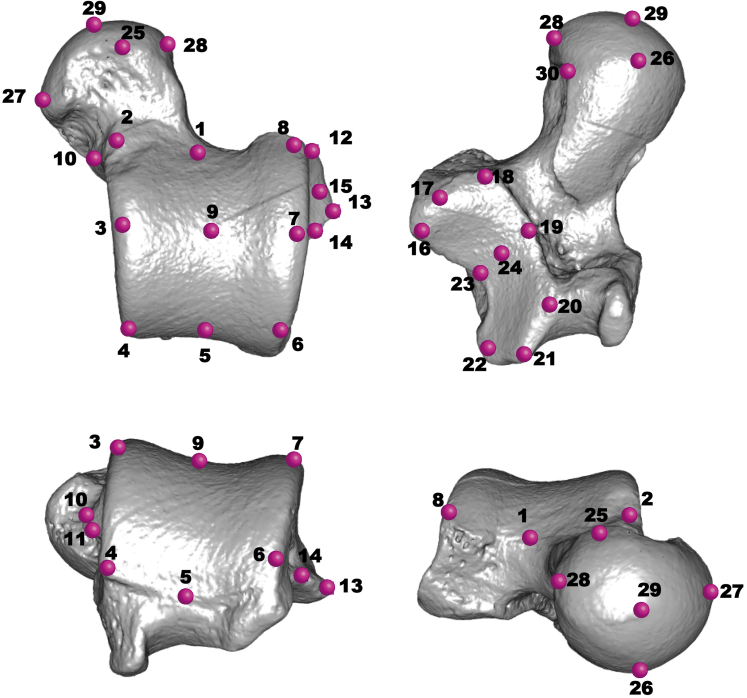
Thirty landmarks in situ illustrated using a talus of *Chiropotes satanas* (AMNH 95760). The talus is visualized in a dorsal, plantar, anterior, and posterior view.

The 3D models of platyrrhine fossils and extant individuals were used to carry out geometric morphometric (GM) analyses. Most of the specimens were right tali, but some of them were reflected when necessary to provide a uniformly right-sided dataset. First, a series of 30 Cartesian coordinates were collected on the surface of the models following the homologous landmark map proposed by Turley and Frost (2013) (Fig. 1). These coordinates were collected using Landmark editor v. 3.6 (Wiley et al., 2005) and then imported into R 3.4.0 (http://www.R-project.org/) to carry out the GM analyses using the ‘geomorph’ package (Adams and Otárola-Castillo, 2013). A Procrustes superimposition was performed on these coordinates, to remove differences due to scale, translation and rotation, thus obtaining shape variables (Bookstein, 1997). Because some of the fossils had missing landmarks due to postdepositional damage (SOM S2), a missing data imputation procedure was performed (Gunz et al., 2009). By using the complete cases from the extant comparative sample, multivariate regression was used to estimate the location of the missing landmarks using the estimate.missing() function in ‘geomorph’ (Adams and Otárola-Castillo, 2013). Here each landmark with missing values was regressed on all other landmarks for the set of complete extant specimens, and the missing landmark values were then predicted by this linear regression model (Gunz et al., 2009). This procedure was carried out to avoid the problem of having different specimens with different missing landmarks. Then, the obtained shape variables were used in a principal component analysis (PCA) to establish initial morphological affinities between all extinct and extant species using the prcomp() function from the ‘stats’ package (R Core Team, 2017).

A canonical variates analysis (CVA) of the extant species was carried out using the shape variables and taxonomic family as a priori category to test whether talar morphology could be used to distinguish between these different taxonomical levels (Tallman and Cooke, 2016). This analysis was carried out using the CVA() function from the R package ‘Morpho’ (Schlager, 2017). Then, using the obtained canonical coefficients, the different fossils were defined within the taxonomical levels to establish possible similarities. Based on the work of Youlatos and Meldrum (2011), the platyrrhine species were classified according to their main locomotion mode in three categories (i.e., clamber/suspensory, leaper/clawed and arboreal quadrupedalism) (Table 1) and another CVA was performed using these categories. This CVA was initially carried out with the extant comparative sample and then, using the obtained canonical coefficients, the different fossils were defined within the proposed locomotion categories. In this way it was possible to have an initial approximation of the possible locomotor repertoires of the fossil specimens, as well as to test if talar shape could be used to distinguish different locomotor habits. The percentage of correct classification of the two performed CVAs was assessed via a jackknife resampling procedure.

Additionally, to visualize morphological affinities between the extant species and the fossils, a morphological affinity dendogram was generated by applying Ward's method for agglomerative-hierarchical cluster analysis, since this algorithm has been recommended for morphometric data (Hammer and Harper, 2008). Euclidean distances were used as the similarity index, and the dendrogram was computed using all the principal components (PCs) from the PCA considering the extant species and the fossils.

Additionally, all the shape changes associated with the proposed analyses were visualized, when necessary, using 3D warpings of the surface models. First one of the surface models closest to the consensus configuration was warped to match the multivariate mean using the thin plate spline method (Bookstein, 1997), then the obtained average model was warped to represent the morphological variation depending on the different analyses performed.

### 2.4 Phylogeny

**Figure 2:**
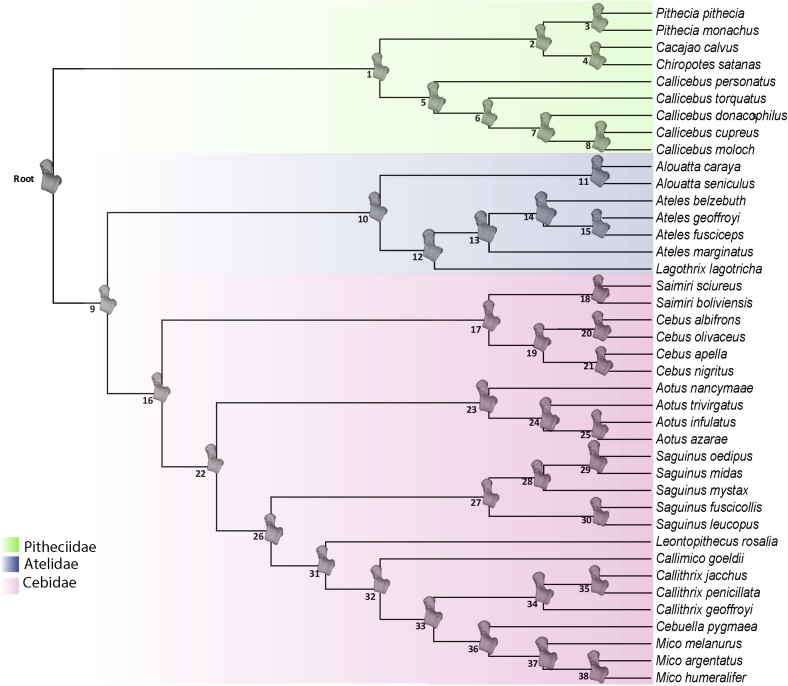
Extant platyrrhine phylogeny used in the present study. Node numbers are displayed. In the nodes, the ancestral shape reconstructions are shown, using the squared-change parsimony approach of Maddison (1991).

An up-to-date platyrrhine phylogeny (Aristide et al., 2015) was modified slightly in Mesquite v. 3.04 (Maddison and Maddison, 2017), adjusting some species names to match those in the morphological dataset, adding some species (*Ateles marginatus*, *Aotus infulatus*, *Chiropotes satanas*, *Mico melanurus*, and *Saguinus leucopus*; Sena et al., 2002, Bonvicino et al., 2003, Araripe et al., 2008, Menezes et al., 2010, Morales-Jimenez et al., 2015) by hand and removing species for which there were no talar data. The resulting phylogeny (Fig. 2; SOM S3) was used to perform all the described comparative analyses.

### 2.5 Locomotor mode percentages

It was necessary to establish if there was a significant association between talar morphology and locomotion to test whether talar morphology is a good proxy for locomotion. First the locomotor mode percentages (LMPs) (i.e., the percentage time a species spends performing a certain locomotor behavior) of 31 platyrrhine species were obtained from Youlatos and Meldrum (2011). This dataset compiled several sources from different publications, and considered five different locomotor behaviors: bridge/suspensory locomotion, arboreal quadrupedal walk, clamber/vertical climb, leap/drop/hop, and clawed locomotion. A PCA of the correlation matrix of the LMPs of the species used in the present study (*n* = 23) was carried out to see if main locomotion modes could be distinguished. The phylogenetic signal of the LMPs was estimated using a mathematical generalization of the K-statistic (Blomberg et al., 2003) appropriate for multivariate data (i.e., Kmult) (Adams, 2014). The K-statistic varies between 0 (no phylogenetic signal in the data as in a star phylogeny) to 1 (data fit a Brownian motion (BM) model of evolution) or significantly more (species are more similar than expected under BM) (Blomberg et al., 2003). Subsequently, both a standard partial least squares (PLS) and a phylogenetic PLS analysis were performed to examine the association between the LMPs and the shape variables of the species that were present in both datasets (Rohlf and Corti, 2000). The standard PLS calculates the degree of covariation between the two datasets, while the phylogenetic PLS also accounts for phylogeny under a BM model of evolution (Adams and Felice, 2014). Partial least squares has the advantage that it does not assume that one set of variables is dependent on the other, thus being a useful tool for assessing the relationship between sets of variables that might covary but for which there is no a priori directional relationship (Rohlf and Corti, 2000). These results were expected to contribute to the understanding of the relationship between talar morphology and locomotion. In addition, the first two PCs of the PCA of the LMPs were used to estimate the ancestral states for internal nodes, first using maximum likelihood and then by interpolating the states along the branches of the tree according to Felsenstein (1985) in the R package ‘phytools’ (Revell, 2012, Revell, 2013). In this way, we tried to reconstruct the ancestral locomotor condition of the NWM using published locomotion data.

### 2.6 Evolutionary modeling

Phylogenetic signal was estimated for talar shape, centroid size and body mass using the Kmult statistic (Adams, 2014). To visualize the phylogenetic relationships in the morphospace, the phylogeny was projected onto the space identified by the first two PCs obtained from the covariance matrix of the average shapes of the analyzed taxa (Klingenberg and Gidaszewski, 2010). In addition, by using the squared-change parsimony approach of Maddison (1991) the ancestral body masses, centroid sizes and shapes (Fig. 2) for the different nodes of the phylogeny were estimated. This approach was preferred because the squared-change parsimony reconstruction has maximum posterior probability under a BM evolutionary model (Maddison, 1991). Therefore, the ancestral reconstructions represent conservative hypotheses about the possible trait values of the actual ancestors.

A multivariate phylogenetic generalized least square regression (PGLS) was used to evaluate the association between shape and some size measures (i.e., body mass and centroid size) to analyze the influence of allometry on talar shape. Even though talar centroid size and body size are highly correlated (R^2^ = 0.94; *p*-value < 0.001), two separate regressions were performed using these two size measures to provide a full picture. By modeling residual variation assuming a BM evolution mode, PGLS takes into account the expected absence of independence across taxa due to phylogenetic structuration, which is expected to affect the covariance in trait values (Adams, 2014). The body mass data were gathered from the available literature (Smith and Jungers, 1997, Aristide et al., 2015). As male and female body mass are highly correlated among the living platyrrhine species, average body mass was used in the analyses (Aristide et al., 2015).

The first five PCs of the extant dataset (63.57% of explained variance) were used in the following comparative analyses based on the results obtained from a broken-stick model used to assess significance of variance (Jackson, 1993). This procedure was performed to reduce the number of variables, given that 40 taxa, each one represented by 30 3D landmarks, were analyzed.

It was tested whether talar morphology exhibited shape convergence between some of the platyrrhine groups by using the SURFACE method implemented as the runSurface() function from the R package ‘surface’ (Ingram and Mahler, 2013). This method fits a model of adaptive radiation in which lineages might experience shifts to adaptive peaks on a macro-evolutionary landscape without reference to a priori hypotheses specifying which lineages correspond to particular peaks (Mahler et al., 2013). Starting with an Ornstein-Uhlenbeck (OU) model in which all species are attracted to a single adaptive peak in trait space (Butler and King, 2004), SURFACE uses a stepwise model selection process based on the finite-samples Akaike information criterion (AICc) to fit increasingly complex multi-peak models (Mahler et al., 2013). In the ‘forward phase’ a new peak shift is added to the branch of the phylogeny that most improves model fit across all traits, and shifts are added until none results in further improvement (i.e., ΔAICc < 2) (Ingram and Mahler, 2013). Then in the ‘backward phase’ the method assesses whether the AICc score is improved further by collapsing regimes in different branches to shift toward shared adaptive peaks rather than requiring each to occupy a unique peak, to identify possible convergence (Mahler et al., 2013). This ‘backward phase’ proceeds step by step until no further improvement is achieved. The SURFACE method can thus survey several hundred OU models, obtaining a model with the highest absolute statistical support among those explored. Importantly, convergence is understood here as described by Ingram and Mahler (2013) as evolution towards the same adaptive peak, therefore distinguishing between convergence occurring as a result of deterministic adaptation to speciﬁc ecological conditions and convergence occurring by chance under simple random-walk processes (Stayton, 2015). SURFACE does not consider the evolutionary correlations among variables, thus being unable to fit data in a multivariate way, therefore the model found by SURFACE was translated into the ‘mvMORPH’ package and tested along diverse alternative hypotheses in order to test if the SURFACE model was also the best adaptive explanation for the evolution of talar shape.

It has been suggested that the talus has been shaped through habitat utilization within specific contexts – both locomotor and ecological – therefore being associated with the adaptive radiation suggested for platyrrhine evolution (Youlatos and Meldrum, 2011). Using the platyrrhine phylogeny and talar shape and size data a series of evolutionary models were tested for congruence with the actual morphological data (Freckleton et al., 2003). Model selection analyses were performed with the ‘mvMORPH’ package for R (Clavel et al., 2015), which allowed fitting several evolutionary models to trait data and a phylogeny in a multivariate framework. For each model, the relative fit was assessed using the AICc (Burnham and Anderson, 2013). Several models were assessed, with BM as the simplest, while more complex models included early burst (EB) (Harmon et al., 2010) as well as several adaptive OU models (Butler and King, 2004). Under BM, trait evolution is simulated as a random walk through trait space, and phenotypic difference between sister taxa is expected to grow proportional to the sum of branch lengths between them (Wilson et al., 2015). Support for a BM model suggests that morphological disparity is uniformly increasing over time. In the EB model, the rates of Brownian evolution decays exponentially with time, thus representing niche-filling scenarios (Harmon et al., 2010). Support for the EB model suggests that most of the morphological disparity present in extant NWM was partitioned early in their evolutionary history and therefore provides weight to the LLH (Harmon et al., 2010). The OU model describes trait evolution under stabilizing selection, whereby there is attraction to a selective optimum; the strength of attraction to this selective optimum (i.e., the strength of selection) is measured using the α parameter (Butler and King, 2004). Several OU models were constructed (SOM S4) to test if adaptive evolution could explain talar shape diversification. Each one of the proposed models represents an alternative biological hypothesis regarding the possible factors that might have influenced the adaptive landscape for platyrrhines. These models were based on different adaptive evolution hypotheses and ecological niches suggested for platyrrhine species (Rosenberger, 1992, Norconk et al., 2009, Youlatos and Meldrum, 2011, Allen and Kay, 2012, Aristide et al., 2015, Aristide et al., 2016). Many of the analyzed models were derived and adapted from the work of Aristide et al., 2015, Aristide et al., 2016, however due to the fact that these models were generated to analyze different traits (i.e., brain shape and body mass), only those that were more general were applied, while others were not considered. In addition, other models specifically designed for talar morphology were generated.

The first multi-peak model contained three separate optima that corresponded to the three platyrrhine families (OU-Clade), while the second was based on data concerning diet composition (OU-Diet Composition) and also had three optima (i.e., average annual percentages of plant parts and insects in the diets of platyrrhine genera) (Norconk et al., 2009). This diet model was considered because access to different diets requires differences in both locomotion and postural repertoire (Rosenberger, 1992). The third (OU-Locomotion A) was defined according to main locomotion categories and had three optima (clamber/suspensory, leaper/clawed and arboreal-quadrupedalism) (Youlatos and Meldrum, 2011). Another locomotor model (OU-Locomotion B) similar to the previous one was tested, however in this one, only *Callimico, Callithrix* and *Cebuella* were considered within the leaper/clawed category, while the rest of the callitrichines were classified as arboreal quadrupeds based on the fact that they exhibited higher percentages of arboreal quadrupedal walking (Youlatos and Meldrum, 2011). Additionally a third locomotor model (OU-Locomotion C) was designed by combining the OU-Locomotion A and the convergence result obtained from the SURFACE method; this model had four optima representing the three locomotor categories already mentioned, as well as one adaptive peak representing the convergence result found by SURFACE.

Following Aristide et al., 2015, Aristide et al., 2016 a multidimensional niche model was defined (OU-Multidimensional Niche) with five optima that combined diet and locomotion information (Rosenberger, 1992). Two other models were generated based on the main canopy level occupied by the different species analyzed. The first one (OU-Canopy A) had three different optima (understory, middle and upper), while the second (OU-Canopy B) had four optima, which were the same as the three previous ones, but included an additional optimum for *Aotus*, which has been observed occupying all canopy levels with relative frequency (Fleagle, 2013). The canopy level classifications were performed using the data available in the Animal Diversity Web (ADW) of the University of Michigan (http://animaldiversity.org/) and Fleagle (2013). Different canopy levels are differentially structured, thus requiring different locomotor behaviors, therefore it was expected that these differences might impact on talar morphology.

It is relevant to bear in mind that these different evolutionary models are generated to help in the understanding of possible underlying evolutionary processes, but they do not necessarily represent complete explanations (i.e., model selection is not an end in itself but a helpful approach in contributing to reasoning about the evolutionary mechanisms that might explain the observed variation in the analyzed traits) (Cressler et al., 2015). The different OU models based on different biological criteria were tested and their relative fit was assessed using AICc scores. In this manner, a measure of the relative explanatory power of each hypothesis (ΔAICc) was obtained. In addition to the OU models based on biological criteria, a single-peak OU model was also tested (if supported, that would suggest that there is a single, optimal talar shape for all of the platyrrhines), as well as a model representing the result obtained from the SURFACE method.

A mean relative disparity-through-time (DTT) plot of the temporal pattern of change in relative talar shape disparity along the platyrrhine phylogeny was calculated using the first five PCs obtained from the shape PCA and also for centroid size (Harmon et al., 2003). Disparity was measured as $D=\;\sum \left(d_{i}\right)/n-1$ where *d*_*i*_ is the pairwise Euclidean distance between species and *n* is the number of species. First, disparity was calculated for the entire platyrrhine clade, and then for each sub-clade. Disparity of each sub-clade was standardized by dividing it by the disparity of the entire clade (relative disparity sensu Harmon et al., 2003). Such analyses allow comparison of the observed pattern of intra-clade versus among-clade disparity through time with a BM expectation. Therefore, high relative disparity values are a sign of extensive within-clade diversification and among-clade overlap, whereas values near 0 might imply that variation is mostly partitioned among clades (Harmon et al., 2003). The ‘geiger’ package for R (Harmon et al., 2008) was used to generate DTT plots.

### 2.7 Body mass

Due to the lack of body mass predictions for the Río Cisnes talus and for *P. marianae*, as well as the absence of robust mass predictions for some of the other fossils, it was decided to include calculation of this relevant biological information for the fossil sample under study. The predicted masses of the fossil taxa were derived from surface area measurements of the talar articular facets taken directly from 3D digital models. Articular surfaces of the talus have proven to be reliable and accurate predictors of body mass across primates, and using 3D surface areas taken directly from digitized models of the fossil has yielded precise and accurate results (Lieberman et al., 2001, Yapuncich et al., 2015). Mass regressions were based on a sample of 123 individual platyrrhine tali from across 15 genera (SOM S5) that were MicroCT scanned at the Shared Materials Instrumentation Facility (SMIF) at Duke University or the Microscopy and Imaging Facility (MIF) at the American Museum of Natural History. The creation of 3D surface models, the measurement of facet surface areas, and the construction of new mass predictive equations follows methods set out in Yapuncich et al. (2015).

**Figure 3:**
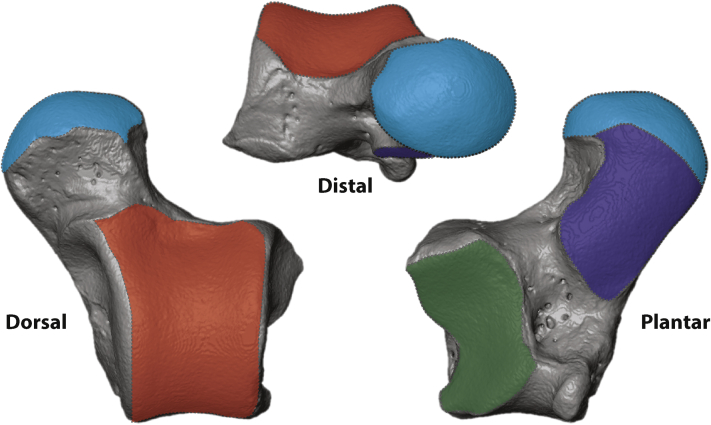
Facet measurements for the talus in dorsal, distal, and plantar orientations. Articular surface areas were measured for the ectal (green), trochlear (red), navicular (light blue) and sustentacular facets (dark blue). Talus measurements shown on *Callimico goeldii* (USNM 395455). (For interpretation of the references to color in this figure legend, the reader is referred to the web version of this article.)

Facet measurements from all 123 individuals were reduced to 40 species-dimorphic average data points; male and female individuals of the same species were all averaged into a single data point unless reported dimorphism levels were above 20%. Taxa with dimorphism levels above this threshold were treated as separate male and female data points for that species. All published body mass data for the dimorphism cutoffs and for the creation of the mass regressions was taken from Smith and Jungers (1997). Body mass data from the literature were regressed onto the averaged facet surface area data to generate four independent body mass estimates from articular surfaces of the talus: the ectal (or posterior calcaneal) facet, navicular facet, sustentacular facet, and trochlear (lateral tibial) facet (Fig. 3). Unlike in the sample of extant tali, the fossil sample did not consistently have all four facets pristinely represented for every individual so an average mass derived from estimates of all intact facets was used for the body mass prediction.

## 3 Results

### 3.1 Morphological affinities

**Figure 4:**
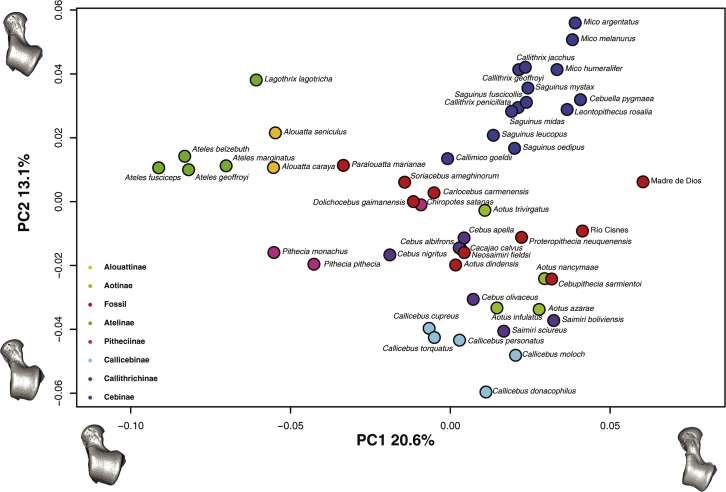
Principal component analysis (PCA) of the talar shape variables (only the two first PCs are shown) including both the extant and fossil samples. One of the models closest to the mean shape was warped to match the multivariate mean using the thin plate spline method. The obtained average model was then warped to represent the variation along the two plotted PC axes in both analyses. Note that *Cacajao calvus* is not miscolored, but *Cebus albifrons* exactly overlays it.

The PCA shows three major regions of occupied shape space (Fig. 4), which tend to correspond to the previously described locomotor categories. Principal component 1 mostly distinguished between the small-bodied Callitrichinae, exhibiting claw-assisted scansorial and clinging positional behaviors towards one extreme of the axis, and the large-bodied Atelidae, exhibiting climbing/clambering and suspensory behaviors with tail-assisted suspension toward the other extreme (Youlatos and Meldrum, 2011). The more derived locomotor behaviors described above were separated from increasingly quadrupedal species on PC2. There was a central cluster of more ‘generalist’ species, which are predominately quadrupedal although they engage in other locomotor behaviors, such as *Chiropotes* and *Cebus*, while the negative extreme of PC2 was occupied by the most quadrupedal species (i.e., *Saimiri* and *Callicebus*). The Pitheciinae, which are located at the center of the plot, are divided between the most quadrupedal species (i.e., *Cacajao* and *Chiropotes*) from those that exhibit more suspensory behaviors (i.e., *Pithecia*), which are located almost at the same position as *Alouatta* along PC1. Interestingly, some *Cebus* species and the Pitheciinae subfamily exhibit the most ‘generalist’ talar morphology. The variation on the negative side of PC1 can be associated with a longer posterior and shorter anterior calcaneal facet, a broader talar head, a lower trochlea, and increased trochlear wedging. These traits have been linked with greater mobility of the subtalar and transverse talar joints, along with a greater range of flexion-extension at the upper ankle joint (Meldrum, 1990). The morphological variation on the positive side of PC1 is related to a relatively increased anterior calcaneal facet and relatively shorter trochlea antero-posteriorly with more parallel lateral and medial rims. These features have been associated with frequent leaping as observed in some callitrichines (Meldrum, 1990). In contrast, PC2 mostly differentiates between decreased dorso-lateral articular surfaces on the positive side of the axis and those showing increased dorso-lateral articular surfaces on the negative side.

Most of the fossil sample is located at the center of the PCA, in an area of the morphospace mostly occupied by locomotor ‘generalist’ species. Only one fossil specimen, the Madre de Dios talus, occupies an area on an extreme of the plot. The oldest Patagonian fossils (*Dolichocebus, Soriacebus* and *Carlocebus*) are located near the center of the PCA, while *A. dindensis* and *N. fieldsi* are located among *Cebus* and *Cacajao*. Río Cisnes and Madre de Dios are located in zones of the morphospace that are not shared with any extant species under analysis. Although on PC2 these specimens are located in the ‘generalist’ area of the morphospace, on PC1 they are unique. *Proteropithecia* occupies a position between the cebids and Río Cisnes, whilst *Paralouatta* occupies a position near *Alouatta*.

**Figure 5:**
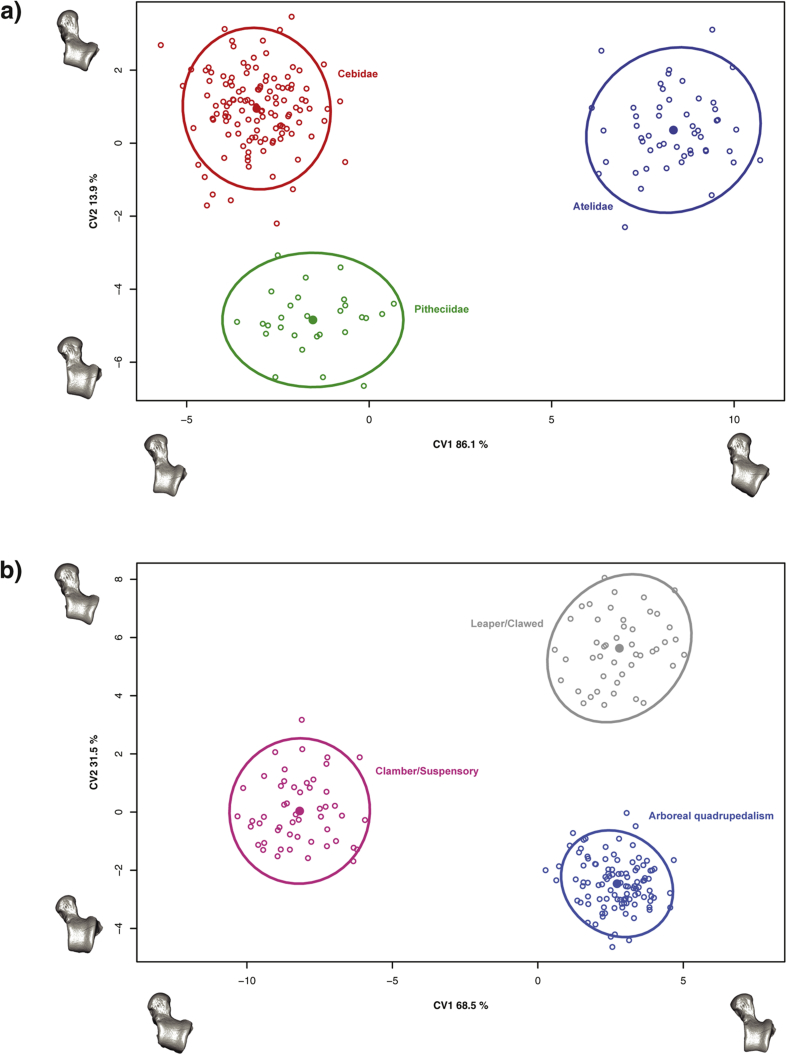
Canonical variate analyses (CVA) of talar shape using a) taxonomic family categories and b) locomotor classifications. The circles represent 90% confidence intervals, while the filled dots correspond to the group means. One of the models closest to the mean shape was warped to match the multivariate mean using the thin plate spline method, then the obtained average model was warped to represent the variation along the two plotted CV axes in both analyses.

The two CVAs showed clear and significant differentiation both among the platyrrhine families and according to locomotion (Table 3 and Fig. 5a and 5b). Consequently, it seems that talar morphology is a good descriptor of taxonomic affiliation at least at the family level, and that its shape reflects different locomotor behaviors. When classified according to the extant platyrrhine families, most of the fossils were classified as members of Cebidae or in some cases as belonging to Pitheciidae. These results are consistent with the PCA that indicated most fossils tend to show an intermediate morphology, most similar to the Pitheciinae and Cebinae subfamilies. This morphology could be interpreted as potentially primitive for platyrrhines. In morphological terms, the shape changes associated with CV1 are a broader and lower trochlear surface with a shorter talar neck on the positive side of the axis, while the negative side is related to a narrower, higher and saddle-shaped trochlea, along with a longer talar neck. A more wedge shaped trochlea lies on the positive side of CV2, while the negative side shows a narrower and higher trochlear surface. The CVA using locomotor categories classified most fossils as arboreal quadrupeds, with only Madre de Dios being classified differently, as leaper/clawed. The morphological changes are broadly similar to the ones described above for the family CVA, especially for CV1, but with the axes inverted.

**Table 3:** Canonical variate analyses results.

a) Extant sample			
Extant sample classification:	% Correctly classified (jacknifed)		
	Family	Locomotion	
	95.57%	98.03%	
Mahalanobis distances among taxonomic families and *p*-values (above the diagonal)	Atelidae	Cebidae	Pitheciidae
Atelidae	0	*p* < 0.0001	*p* < 0.0001
Cebidae	11.4336	0	*p* < 0.0001
Pitheciidae	11.1636	5.9898	0

Mahalanobis distances among locomotor categories and *p*-values (above the diagonal)	Leaper/clawed	Clamber/suspensory	Arboreal quadrupedalism
Leaper/clawed	0	*p* < 0.0001	*p* < 0.0001
Clamber/suspensory	12.3204	0	*p* < 0.0001
Arboreal quadrupedalism	7.9371	11.1666	0

b) Fossil sample				
	Obtained classification	Posterior probabilities		
	Family	Atelidae	Cebidae	Pitheciidae
*Dolichocebus gaimanensis*	Cebidae	0.00000006	0.99999994	0.00000000
Madre de Dios	Cebidae	0.00000000	0.99999999	0.00000001
Río Cisnes	Cebidae	0.00000000	0.99994768	0.00005232
*Cebupithecia sarmientoi*	Cebidae	0.00000000	0.99999257	0.00000743
*Carlocebus carmenensis*	Cebidae	0.00000000	0.99999257	0.00000000
*Soriacebus ameghinorum*	Pitheciidae	0.00000000	0.03667571	0.96332429
*Proteropithecia neuquenensis*	Cebidae	0.00000000	0.72229885	0.27770115
*Neosaimiri fieldsi*	Cebidae	0.00000000	0.99999257	0.00000000
*Aotus dindensis*	Pitheciidae	0.00000000	0.03768954	0.96231046
*Paralouatta marianae*	Cebidae	0.00000000	0.99999999	0.00000001
	Locomotion	Leaper/clawed	Clamber/suspensory	Arboreal quadrupedalism
*Dolichocebus gaimanensis*	Arboreal quadrupedalism	0.062085723	0.024260237	0.913654040
Madre de Dios	Leaper/clawed	0.999883487	0.000000000	0.000116513
Río Cisnes	Arboreal quadrupedalism	0.000000003	0.000000000	0.999999997
*Cebupithecia sarmientoi*	Arboreal quadrupedalism	0.000000034	0.000000000	0.999999966
*Carlocebus carmenensis*	Arboreal quadrupedalism	0.000000010	0.000000000	0.999999990
*Soriacebus ameghinorum*	Arboreal quadrupedalism	0.000000013	0.000000000	0.999999987
*Proteropithecia neuquenensis*	Arboreal quadrupedalism	0.000000084	0.000000000	0.999999916
*Neosaimiri fieldsi*	Arboreal quadrupedalism	0.002491686	0.000002963	0.997505351
*Aotus dindensis*	Arboreal quadrupedalism	0.000000153	0.000000000	0.999999847
*Paralouatta marianae*	Arboreal quadrupedalism	0.004193355	0.000000000	0.995806645

**Figure 6:**
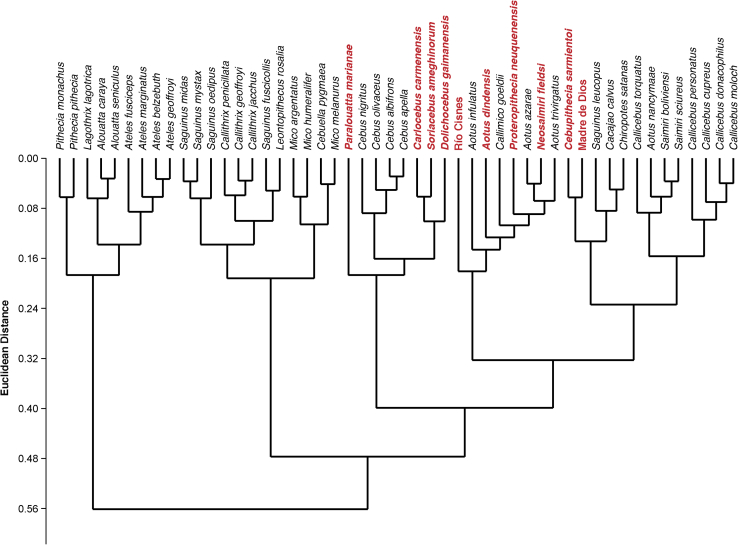
Hierarchical clustering analysis of shape PCs using Ward's method. Fossils are in bold and red, while extant species are in black. (For interpretation of the references to color in this figure legend, the reader is referred to the web version of this article.)

The agglomerative-hierarchical cluster analysis of the PCs using Ward's method showed the morphological affinities between extant species and the fossils (Fig. 6). Three main clusters are easily noticeable, one comprising the most suspensory species (i.e., the Atelidae and *Pithecia*), another consisting of most of the Callithrichinae (excepting *Callimico* and *S. leucopus*), and another one containing all the fossil specimens and mostly arboreal quadrupedal and locomotor ‘generalist’ species (e.g., *Saimiri*, *Callicebus*, *Aotus* and *Cebus*). This analysis revealed that most fossils are relatively similar, clustering in certain groups within this locomotor ‘generalist’ and arboreal quadrupedal cluster. For instance, *C. carmenensis*, *Soriacebus* and *Dolichocebus* clustered together with *Cebus* and *Paralouatta. N. fieldsi, A. dindensis, P. neuquenensis* and Río Cisnes clustered within a group comprising *Callimico* and most of *Aotus*, whilst *Cebupithecia* clustered together with Madre de Dios in a group consisting of *S. leucopus*, *Cacajao, Chiropotes, Callicebus* and *Saimiri.*

### 3.2 Locomotor mode percentages

**Figure 7:**
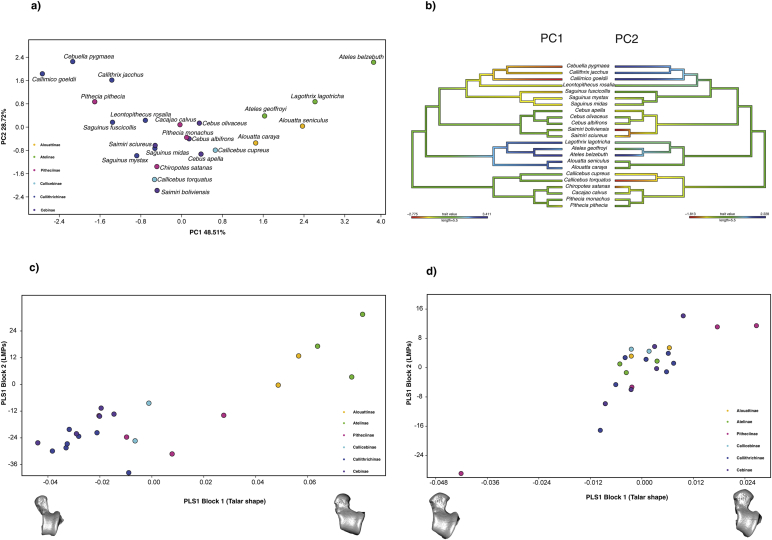
a) Principal component analysis (PCA) of the LMPs (i.e., bridge/suspensory locomotion, arboreal quadrupedal walk, clamber/vertical climb, leap/drop/hop, and clawed locomotion); b) PC1 (left) and PC2 (right) values of the LMPs for each species mapped on the phylogeny, the values at nodes and branches were reconstructed using a maximum-likelihood ancestral character estimation method based on a Brownian motion model of evolution; c) depicts the standard partial least squares (PLS) and d) the phylogenetic PLS analysis of the LMPs and the shape variables. One of the models closest to the mean shape was warped to match the multivariate mean using the thin plate spline method, then the obtained average model was warped to represent the covariation between the two blocks of data for PLS1.

Locomotor mode percentages showed a significant phylogenetic signal (Kmult: 0.54; *p*-value: 1e-04; 10,000 permutations). In a similar fashion to the shape PCA, the PCA of the LMPs showed a clear distinction along PC1 between the suspensory species (i.e., atelids) and those exhibiting leaping and vertical clinging (i.e., callitrichines). Principal component 2 distinguished mainly the most quadrupedal species (i.e., *Callicebus* and *Saimiri*) from species with other locomotor behaviors (Fig. 7a). At the center of the plot there is an overlap of ‘generalist’ quadrupedal species that also exhibit other locomotor behaviors, although less frequently. Interestingly, *Pithecia pithecia* is located next to Callitrichinae due to its frequent leaping behaviors (Walker, 2005), in contrast to the talar shape PCA where it is located relatively near suspensory species on PC1. The LMPs also showed a strong and significant covariation with talar shape (r-PLS: 0.84; *p*-value: 0.0022; 10,000 permutations), as well as when accounting for the phylogenetic structure of the data (phylogenetic r-PLS: 0.87; *p*-value: 0.0014; 10,000 permutations) (Fig. 7c and 7d, respectively), thus establishing that there is a robust association between talar shape and locomotor behavior. The PC loadings and PLS singular vectors for the locomotor mode percentages are provided in SOM S6. The PC1 of the LMP values for each species, mapped on the phylogeny using a maximum-likelihood ancestral character estimation method based on a BM model of evolution, showed results consistent with the previously mentioned analyses. The ancestral state was reconstructed as arboreal quadrupedalism, while both suspension and leaping/clawed locomotion are derived locomotor behaviors (Fig. 7b). The ancestral state reconstruction for the PC2 of the LMPs showed a distinction between the most quadrupedal species and the other locomotor behaviors (Fig. 7b).

### 3.3 Evolutionary modeling

**Figure 8:**
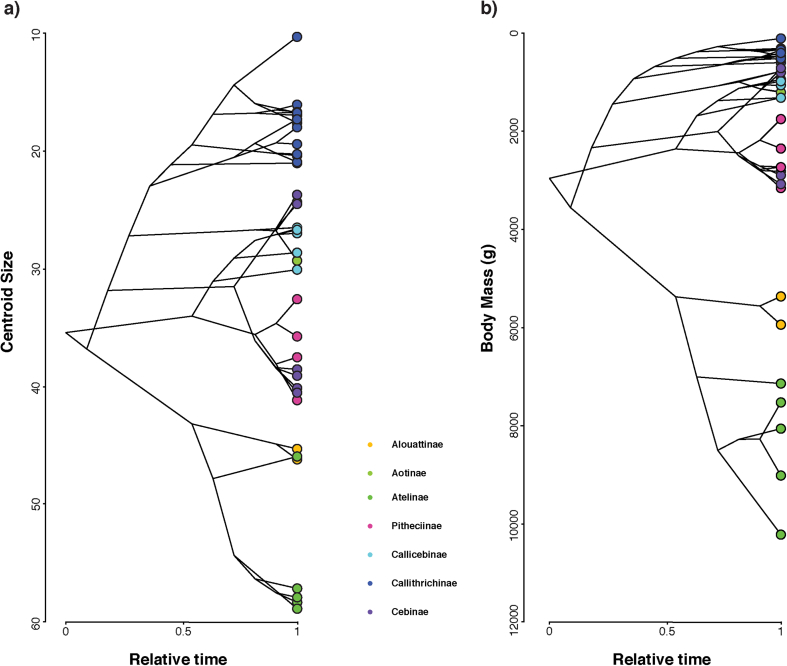
Traitgram of a) talar centroid size and b) body mass of the 40 extant platyrrhine species considered here. Both body mass (K: 3.09; *p*-value: 1e-04; 10,000 permutations) and centroid size (K: 3.03; *p*-value: 1e-04; 10,000 permutations) showed significant phylogenetic signals.

**Figure 9:**
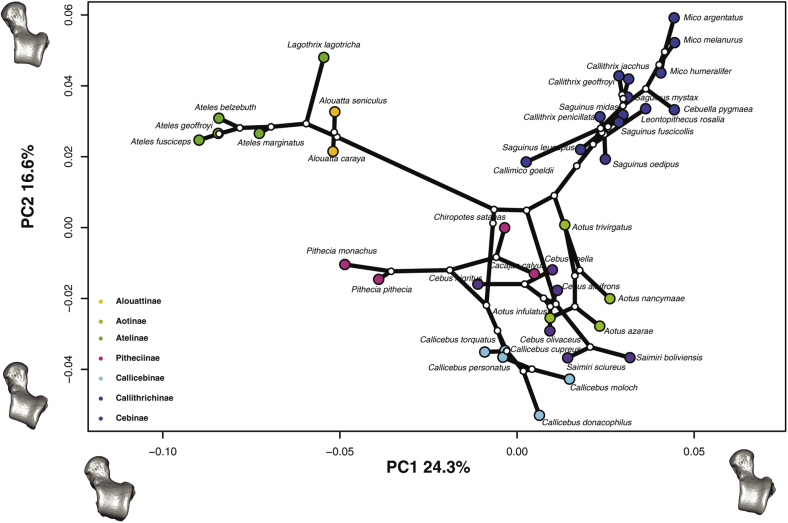
Phylomorphospace of the extant platyrrhine sample (only the first two PCs are shown). One of the models closest to the mean shape was warped to match the multivariate mean using the thin plate spline method, then the obtained average model was warped to represent the variation along the two plotted PC axes in both analyses.

Phylogenetic signal was found for shape (Kmult: 0.46; *p*-value: 1e-04; 10,000 permutations), centroid size (K: 3.03; p-value: 1e-04; 10,000 permutations), and body mass (K: 3.09; *p*-value: 1e-04; 10,000 permutations). The obtained traitgrams showed that early on during platyrrhine evolution there is a strong divergence in size, particularly for the large-bodied Atelidae (i.e., talar centroid size and body mass) (Fig. 8a and 8b). The ancestral platyrrhine at the root of the phylogeny was reconstructed as a medium-sized monkey (body mass: 2966 g; 95% LCI: 1623 g; UCI: 4309 g), with a talar centroid size similar to *Pithecia monachus* (centroid size: 35 mm; 95% LCI: 29 mm; UCI: 41 mm). The phylomorphospace (Fig. 9.) shows an almost total absence of overlap between major phylogenetic branches, thus suggesting that there is no evident convergence in talar shape among the main platyrrhine clades. Nonetheless, there is some overlap in the negative side of PC2 between mostly arboreal quadrupedal species. Interestingly, the best model found by the SURFACE method exhibited six different adaptive regimes, with one of them convergent between *Callicebus* and *Saimiri*, thus suggesting a possible convergent scenario for talar shape for these genera (SOM S7). These same genera showed the most negative values in Figure 7b, thus also suggesting possible convergence. In addition these two genera are closely located in the phylomorphospace (Fig. 9), which could indicate a possible convergence, although further analyses are required. It is also important to consider that the SURFACE method used five PCs, while the phylomorphospace displays only the first two axes, so it is possible that convergent features between *Saimiri* and *Callicebus* are more evident when considering more aspects of variation. The phylomorphospace also shows that the main platyrrhine lineages occupy the three major locomotor regions already mentioned for the PCA.

The broken stick model applied to assess the significance of variance of the PCA of the extant sample showed that only the first five PCs had eigenvalues larger than the values randomly generated by the model. These five PCs accounted for 63.57% of the total variance of the sample, thus providing a reasonable approximation of the total amount of talar shape variation. The PGLSs showed that there was a weak but significant association between the first five PCs and centroid size (R^2^: 0.058; F: 2.35; *p*-value: 0.002; 10,000 permutations) and body mass (R^2^: 0.064; F: 2.61; *p*-value: 0.001; 10,000 permutations). Nonetheless, the association is extremely weak; therefore talar shape variation cannot be merely attributed to evolutionary allometric effects.

Several evolutionary models were tested to understand the evolutionary history of both talar shape and centroid size. The overall fit of these evolutionary models is shown in Table 4. For the shape data, the OU-Clade model was the best supported, showing an Akaike weight much higher than any of the other alternative models. This model has three adaptive peaks for each of the three platyrrhine families. For the centroid size data the best supported model was the OU multidimensional-niche hypothesis (Rosenberger, 1992). It is important to bear in mind that one limitation regarding the applied approach is the possible lack of power to detect complex OU models in a multivariate fashion when using many variables (e.g., five PCs) and a relatively small sample (e.g., 40 species). Different evolutionary processes determined the number of species in a particular clade of interest (in the present case 40) therefore there is an intrinsic natural limit to the complexity of the models that can be fit to these systems (i.e., ratio between parameters and sample size). Consequently caution is required when interpreting this analysis because some of the most complex OU models might have performed poorly due to the above limitation and not because they are biologically irrelevant.

**Table 4:** Results of macroevolutionary models fit to shape (five PCs) and centroid size data.

Variable	Shape					Centroid Size				
Model^a^	LogL	Number of parameters	AICc	ΔAICc	Akaike weight	LogL	Number of parameters	AICc	ΔAICc	Akaike weight
BM	446.4964	20	−848.3	12.693077	0.00	−156.5986	2	317.5215	13.214472	0.00
OU1	170.3437	35	−322.6593	538.333781	0.00	−153.0637	3	312.7942	8.487174	0.01
EB	446.2441	21	−845.2973	15.695827	0.00	−156.5986	3	319.8638	15.556814	0.00
OU Clade	488.9381	45	−860.9931	0	0.87	−151.0611	5	313.8869	9.579939	0.01
OU Diet Composition	486.1226	45	−855.362	5.631088	0.05	−149.0932	5	309.951	5.644053	0.05
OU Locomotion A	483.4993	45	−850.1156	10.877499	0.00	−151.8403	5	315.4453	11.138277	0.00
OU Locomotion B	480.5911	45	−844.2991	16.693992	0.00	−152.3345	5	316.4337	12.126677	0.00
OU Locomotion C	483.62	50	−833.0119	27.98118	0.00	−151.8807	6	318.3068	13.999845	0.00
OU Multidimensional Niche	491.7533	55	−830.7289	30.264228	0.00	−143.4035	7	304.307	0	0.91
OU SURFACE	499.674	60	−826.6861	34.306953	0.00	−148.6272	8	317.8995	13.592512	0.00
OU Canopy A	494.7928	45	−855.3575	5.63561	0.05	−149.9652	5	314.4759	10.168915	0.01
OU Canopy B	485.1215	50	−853.3599	7.633165	0.02	−152.0587	6	315.8821	11.575105	0.00

a BM = Brownian motion; OU = Ornstein-Uhlenbeck; EB = Early Burst; models and other abbreviations described in text.

**Figure 10:**
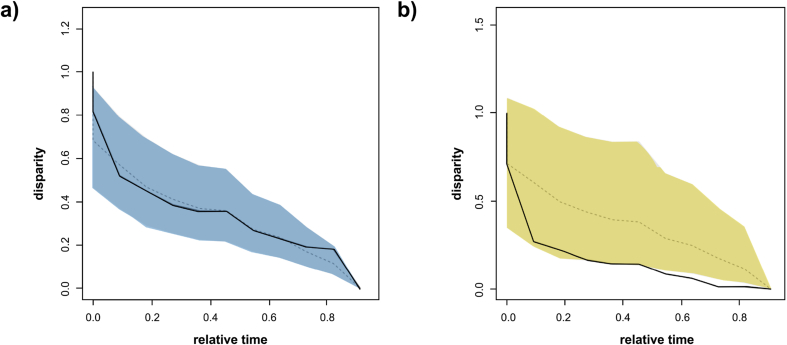
Disparity-through-time (DTT) plots for a) talar shape (i.e., first five PCs) and b) centroid size. Relative disparity at each point indicates the average extant disparity of the sub-clades that had an ancestor at that time with respect to the whole clade disparity. The dashed line represents the expectation under a BM model of evolution (estimated through simulations), while the colored shadow depicts its 95% confidence interval.

Figure 10 shows the DTT plots for a) shape and b) centroid size. The morphological disparity index (MDI) was used to assess the obtained results and it is defined as the area between the observed DTT curve and the median of the simulated DTT curves (Harmon et al., 2003). The shape data seem to follow what is expected under a BM model of evolution (MDI: 0.005), thus suggesting that variation is mainly partitioned according to Brownian expectation (i.e., as expected given platyrrhine phylogeny). On the other hand, centroid size (MDI: −0.181) indicates that the average sub-clade disparity along platyrrhine evolution is lower than expected under a BM. Values drop almost to zero from the early divergence of the platyrrhines, exhibiting minimal variation over time, thus suggesting that most size variation appears among the main NWM sub-clades. The observed pattern is suggestive of an early adaptive radiation due to a niche-filling scenario.

### 3.4 Body mass prediction

All relevant statistics for each of the body mass regressions are reported in Table 5. As previously explained, the fossil sample did not consistently have all four facets represented for every individual so an average body mass estimate was computed (Table 6). All fossils had at least two, and as many as four, facets from which to derive an average mass estimate. Estimates for each individual facet with 95% confidence intervals are also provided in Table 6.

**Table 5:** Relevant statistics for body mass regressions.^a^

Regression statistics (*n* = 40)							
Facet	R^2^	% SEE	Slope (m)	Slope 95% CI	Intercept (b)	Int. 95% CI	QMLE
Ectal	0.958	26.32	1.223	(1.139, 1.307)	3.308	(3.014, 3.601)	1.028
Trochlear	0.961	25.11	1.243	(1.161, 1.325)	2.189	(1.836, 2.541)	1.025
Navicular	0.964	24.28	1.274	(1.193, 1.356)	2.643	(2.329, 2.956)	1.024
Sustentacular	0.950	29.13	1.299	(1.201, 1.397)	2.997	(2.652, 3.343)	1.033

a SEE = standard error of estimate; CI = confidence interval; QMLE = Quasi-Maximum Likelihood Estimator.

**Table 6:** Estimates for each individual facet with 95% confidence intervals (CI) and body mass average estimates.

Genus	Species	Specimen ID	Facet^a^	Mass (g)	Mass (g) 95% CI
*Neosaimiri*	*fieldsi*	IGMKU 89030	Ectal	–	–
			Trochlea	–	–
			Sust.	823	(448, 1510)
			Nav.	694	(413, 1165)
			**Average**	**759**	
*Neosaimiri*	*fieldsi*	IGMKU 89031	Ectal	717	(410, 1250)
			Trochlea	838	(492, 1427)
			Sust.	816	(444, 1498)
			Nav.	755	(450, 1266)
			**Average**	**781**	
*Neosaimiri*	*fieldsi*	IGMKU 89199	Ectal	–	–
			Trochlea	–	–
			Sust.	667	(362, 1226)
			Nav.	1077	(643, 1801)
			**Average**	**872**	
*Aotus*	*dindensis*	IGM 8802	Ectal	651	(373, 1137)
			Trochlea	933	(548, 1586)
			Sust.	881	(480, 1616)
			Nav.	1029	(614, 1721)
			**Average**	**874**	
*Carlocebus*	*carmenensis*	MACN304	Ectal	2667	(1533, 4635)
			Trochlea	2903	(1707, 4934)
			Sust.	2988	(1630, 5476)
			Nav.	3096	(1849, 5183)
			**Average**	**2914**	
*Carlocebus*	*carmenensis*	MACN271	Ectal	–	–
			Trochlea	–	–
			Sust.	2655	(1449, 4862)
			Nav.	2364	(1413, 3952)
			**Average**	**2509**	
*Carlocebus*	*carmenensis*	MACN368	Ectal	1543	(888, 2680)
			Trochlea	–	–
			Sust.	2211	(1208, 4046)
			Nav.	–	–
			**Average**	**1877**	
*Carlocebus*	*carmenensis*	MACN396	Ectal	–	–
			Trochlea	2579	(1517, 4381)
			Sust.	3080	(1680, 5644)
			Nav.	2752	(1644, 4603)
			**Average**	**2803**	
*Soriacebus*	*ameghinorum*	MACN397	Ectal	1429	(822, 2482)
			Trochlea	1981	(1167, 3363)
			Sust.	1687	(921, 3085)
			Nav.	1787	(1069, 2986)
			**Average**	**1721**	
*Dolichocebus*	*gaimenensis*	MACN362	Ectal	1520	(874, 2639)
			Trochlea	–	–
			Sust.	1681	(919, 3076)
			Nav.	–	–
			**Average**	**1601**	
Madre de dios	–	MUSM 2204	Ectal	298	(168, 527)
			Trochlea	–	–
			Sust.	375	(201, 695)
			Nav.	384	(226, 648)
			**Average**	**352**	
*Paralouatta*	*marianae*	MNHNCu 76.3059	Ectal	5029	(2877, 8788)
			Trochlea	5071	(2969, 8662)
			Sust.	4026	(2191, 7397)
			Nav.	–	–
			**Average**	**4709**	
*Proteropithecia*	*neuquenensis*	MLP91lX1	Ectal	1647	(948, 2861)
			Trochlea	2038	(1200, 3459)
			Sust.	2291	(1251, 4192)
			Nav.	2050	(1226, 3425)
			**Average**	**2006**	
Rio Cisnes	–	SGO.PV_974	Ectal	1020	(586, 1773)
			Trochlea	1573	(926, 2670)
			Sust.	2122	(1159, 3882)
			Nav.	1325	(792, 2215)
			**Average**	**1510**	
*Cebupithecia*	*sarmientoi*	UCMP_38762	Ectal	1438	(827, 2497)
			Trochlea	1533	(903, 2603)
			Sust.	2961	(1615, 5426)
			Nav.	1368	(818, 2287)
			**Average**	**1825**	

a Nav. = navicular; Sust. = sustentacular.

The final average estimates are, on the whole, consistent with previously published mass estimates for these fossils based on a variety of different regression methods (Conroy, 1987, Kay et al., 1998, Kay et al., 2008, MacPhee and Meldrum, 2006, Cooke et al., 2011, Youlatos and Meldrum, 2011, Marivaux et al., 2012).

## 4 Discussion

Understanding the evolution of the platyrrhine talus is relevant not only because its morphology has been associated with locomotor behaviors (as confirmed here with the PLS analyses) but also because it is one of the few anatomical structures available in many of the oldest platyrrhine fossils (Youlatos and Meldrum, 2011). The present study contributes to a better understanding of the evolution of this structure. Talar shape shows a significant phylogenetic signal, which indicates that closely related species tend to show similar trait values due to common ancestry. However, at the same time it was found that talar shape significantly covaries with locomotor behavior as measured in LMPs, and thus its morphology can be used to infer some aspects of locomotor repertoire. The modeling analyses found that the phylogenetic hypothesis was the best model to explain talar shape evolution in platyrrhines, while talar centroid size diversification was characterized by an early differentiation related to a multidimensional niche model, in a similar fashion as found for body mass (Aristide et al., 2015). It might seem intriguing that in spite of the high covariation between talar shape and locomotion, the different locomotor models were not the best explanation of talar shape evolution.

One possible reason for this disagreement could be the lack of power to detect complex OU models in a multivariate fashion when using many variables (e.g., five PCs) and a relatively small sample (e.g., 40 species). At least applying current approaches, there is an intrinsic natural limit to the complexity of the models that can be fit to this kind of systems, which is determined by the number of species under analysis. In the present study the most complex models for talar shape (e.g., OU-SURFACE) far exceed the sample size under the study, thus having less power to detect a possibly significant pattern, as compared to simpler models, due to the high number of parameters involved. In spite of this limitation, the simpler analyzed locomotion models (i.e., OU-Locomotion A and B) have the same number of parameters as the model with the highest support (i.e., OU-Clade), therefore at least for the simpler OU models, parameter number does not account for the observed disagreement. It is important to keep in mind that in spite of the inherent limitations of these different evolutionary models, they allow to test different possible evolutionary processes that could explain the observed trait variation. Even though they represent simplified scenarios, by testing them it is possible to quantitatively assess different proposed hypotheses that could explain the diversity of the traits under analysis. In addition, it is also important to consider that the PLS analyses maximize the covariation between two blocks of data, without providing the underlying cause for the observed covariance, while the model-fitting approach tested a series of evolutionary models for congruence with the actual morphological data in order to provide a possible explanation about the underlying causes explaining the observed talar shape and size diversity. Therefore, it is possible that the phylogenetic model might be combining locomotion and other factors that could account for shape differentiation because it is well-known that the distinct behavioral, morphological and ecological adaptations seen in NWM are broadly correlated to specific phylogenetic groups (Ford and Davis, 1992, Rosenberger, 1992, Fleagle and Reed, 1996, Fleagle et al., 1999, Rosenberger, 2002, Youlatos, 2004, Rosenberger et al., 2009). Interestingly, it was found that even though there is a significant association between shape and size, it is quite weak when accounting for phylogeny. Finally, the ancestral NWM was reconstructed as a medium-sized (∼3000 g) arboreal quadruped with generalized talar morphology, consistent with the primitive talar morphology observed in most fossils.

### 4.1 Morphological affinities

Principal component 1 clearly distinguished between species with adaptations for suspensory/climbing behavior from species exhibiting frequent leaping/vertical clinging. The mixture of traits observed for the most suspensory species (i.e., broader head, greater trochlear wedging, a lower trochlea and a shorter anterior and longer posterior calcaneal facet) has been associated with greater mobility of the subtalar and transverse tarsal joints, along with conjoint rotation of the upper ankle joint and a greater range of flexion-extension, which has been related to the flexibility necessary during climbing (Meldrum, 1990). The talar morphology at the other extreme of PC1 can be described by an anteroposteriorly shorter trochlea with more parallel medial and lateral rims and a longer anterior calcaneal facet. These features have been associated with the frequent leaping behavior observed in callitrichines (Youlatos and Meldrum, 2011). In contrast PC2 mainly distinguished between the combination of atelids and callitrichines (i.e., most derived locomotor behaviors) and the more arboreal quadrupedal forms, which can themselves be separated between more ‘generalist’ shapes (i.e., more similar to the fossils such as *Cebus* and the Pitheciinae) and morphologies showing increased dorso-lateral surfaces such as those observed in *Callicebus* and *Saimiri*. Most fossils occupied central positions in the morphospace, exhibiting principally generalized morphologies. These generalized talar shapes could be perhaps related to lower frequencies engaging in more specialized locomotor behaviors, which were probably not common among most Miocene specimens. Interestingly, the Madre de Dios specimen exhibited the most distinct morphology, occupying a region of the morphospace, which is not occupied by any extant species. This unique morphology could perhaps represent a distinctive locomotor repertoire not observed in any extant species, however further analyses are required to test this hypothesis.

### 4.2 Morphological affinities of the analyzed NWM fossils

The oldest platyrrhine fossil with well-described postcranial elements is *D. gaimanensis* from the Sarmiento Formation, Chubut Province, Argentina (Kay et al., 2008). There is still disagreement regarding the phyletic position of this species, and different interpretations have been proposed (Kay et al., 2008, Kay and Fleagle, 2010, Rosenberger, 2010). Based on a series of apparent cranial and postcranial synapomorphies, the LLH perspective states that these fossils are an early member of the lineage leading to modern *Saimiri* (Reeser, 1984, Gebo and Simons, 1987, Tejedor, 2008, Rosenberger et al., 2009, Rosenberger, 2010). The SPH view characterizes this fossil and others as stem platyrrhines, relying mostly on a large cranio-dental parsimony analysis (Meldrum, 1993, Kay et al., 2008, Hodgson et al., 2009, Kay and Fleagle, 2010). The only postcranial element that has been ascribed to *D. gaimanensis* is the well-preserved talus analyzed here, which has been traditionally described as morphologically similar to *Saimiri*, *Cebus*, and *Callicebus*. However, it has also been described as lacking some of the most conspicuous platyrrhine features (Reeser, 1984, Gebo and Simons, 1987, Ford, 1988, Ford, 1990, Meldrum, 1990). The present analyses showed that the talar morphology of *D. gaimanensis* is quite generalized in the morphospace illustrated in Figure 4, which may suggest a combination of characters that are primitive amongst Platyrrhini; according to the CV scores it would be classified as a member of Cebidae. As previously pointed out, some species of *Cebus*, as well as some pitheciids, show a ‘generalist’ talar shape, so this resemblance might be attributed to a conserved morphology. The clustering analysis located this specimen next to *Soriacebus*, *Carlocebus, Cebus* and *Paralouatta* suggesting again that the oldest fossil individuals exhibit a similar primitive morphology. It is interesting that *Cebus* clustered with the oldest analyzed fossils, which could be due to the already mentioned ‘generalist’ morphology. Based on semicircular canal data, *D. gaimanensis* has been described as being relatively agile with medium scores similar to the one observed in cebids (Ryan et al., 2012). The present analyses are consistent with these data, indicating that *D. gaimanensis* was most likely an arboreal quadruped based on the results obtained in the CVA. Its morphology indicates a generalized function with a preponderance of frequent arboreal quadrupedal activities (Meldrum, 1993). The body mass estimate is 1600 g, which is similar to previous estimates based on dentition (i.e., 1500 g; Kay et al., 2008) and to extant platyrrhines such as *Pithecia pithecia.*

*Carlocebus* is the other NWM from Pinturas, although it is evidently larger than *Soriacebus* (Tejedor, 2005b). Its teeth exhibit a more generalized morphology that is thought to be most similar to the Callicebinae (Fleagle and Tejedor, 2002), although some have interpreted this resemblance as homoplasic or primitive. Proponents of the SPH relate *C. carmenensis* to an earlier platyrrhine radiation more closely related to *D. gaimanensis* (Kay et al., 2008). Luckily, there are four well-preserved tali ascribed to *Carlocebus,* thus allowing some degree of intra-specific variability (Meldrum, 1990). These tali have been described as similar to *Saimiri* or Callitrichinae, due to their moderately low and broad trochlea, a very broad, slightly medially directed talar neck, and a broad shallow posterior calcaneal facet (Meldrum, 1990). The present analyses suggest that *Carlocebus* also shows a generalized talar morphology (Fig. 4), similar to *Dolichocebus* and *Soriacebus*. The CVA analysis indicates a morphological affinity with Cebidae. In terms of locomotion, *Carlocebus* is believed to have used a combination of quadrupedal activities with some moderate leaping and/or clambering (Ford, 1990, Meldrum, 1990). The present analyses generally support this view, suggesting mostly arboreal quadrupedal activities. This positional behavioral profile is congruent with its reconstructed paleo-environment and proposed frugivorous diet (Youlatos and Meldrum, 2011). The obtained body mass predictions for the four *Carlocebus* tali range between 1877 and 2913 g, which is consistent with previously published estimates (i.e., 2500 g; Fleagle and Tejedor, 2002) and is similar to extant genera such as *Cebus* or *Chiropotes.*

*Soriacebus ameghinorum* was found in the Pinturas formation and was initially described as having resemblances to Callitrichinae and Pitheciinae (Luchterhand et al., 1986), later being classified as an early member of the latter group (Rosenberger et al., 1990, Rosenberger, 1992, Tejedor, 2008). Nonetheless, as with the rest of the older platyrrhine fossils, it has also been defined as a stem NWM (Kay, 1990, Kay et al., 2008, Kay and Fleagle, 2010). The single available talus analyzed here has been portrayed as resembling those of *Alouatta* and *Pithecia* (Meldrum, 1990). The present analysis indicates that *S. ameghinorum* exhibits an ancestral talar morphology similar to *Dolichocebus* and *Carlocebus,* which are among the oldest Miocene fossils. The analyses carried out to reconstruct its locomotor behavior indicate that it was most likely an arboreal quadruped. It is still debated if the relative talar morphology affinities between *S. ameghinorum* and the pitheciines indicate phylogenetic affinity or homoplasy (Youlatos and Meldrum, 2011). Another possibility is that *S. ameghinorum* exhibits an ancestral morphology that was conserved in the pitheciine lineage. The average body mass estimate for this fossil was 1720 g, thus being similar to previous dental estimates (i.e., 1800 g; Fleagle and Tejedor, 2002) and comparable to the body mass of extant NWM such as *P. pithecia.*

The Madre de Dios talus found in Peruvian Amazonia represents the first early Miocene platyrrhine from northern South America (Marivaux et al., 2012), although recent findings have provided more specimens from the late Miocene of the Peruvian Amazonia belonging to two distinct Cebidae (Marivaux et al., 2016b). In addition to these discoveries, the Peruvian Amazonia has recently provided interesting new findings that contribute to the understanding of early platyrrhine evolution (Bond et al., 2015, Marivaux et al., 2016a, Marivaux et al., 2016b). The discovery of *P. ucayaliensis* from the latest Eocene or Early Oligocene (Bond et al., 2015) and *C. amazonensis* (Marivaux et al., 2016a) from the Late Oligocene, clearly indicates that platyrrhines were well-established in the Amazonian Basin early, thus confirming the expected distribution of NWM in the Neotropics (Marivaux et al., 2016a, Marivaux et al., 2016b). Given that the Madre de Dios talus is a rare example of the NWM postcranial fossil record in Peruvian Amazonia, analyzing it is highly relevant. The talus has not been taxonomically assigned, but has been described as displaying a mixture of talar characteristics mainly found among the Cebidae, and more specifically in the Cebinae (Marivaux et al., 2012). Nonetheless, what is remarkable about this specimen is its reduced size that is most similar to that of the marmosets and tamarins (Cebidae, Callitrichinae). The Madre de Dios talus has been described as being a tiny *Saimiri*-like cebine that was primarily an arboreal quadruped, but also engaged in frequent horizontal leaping and vertical clinging (Marivaux et al., 2012). The analyses performed in this paper showed that the Madre de Dios talus exhibits a particularly distinct morphology. The PCA showed Madre de Dios occupying a region of the morphospace not occupied by any other specimen, which could be related to its particular combination of traits. Interestingly, Madre de Dios clusters with *Cebupithecia* and within a group also comprising *Cacajao*, *Chiropotes* and *S. leucopus*. The CVA using platyrrhine families as categories classified Madre de Dios within the Cebidae, while the locomotion CVA categorized it as the only fossil classified as leaper/clawed. Madre de Dios seems to combine in its morphology some more primitive aspects common to all the analyzed fossils, with some derived characters similar to some members of the Callitrichinae. The evidence thus suggests that Madre de Dios seems to be a small-sized cebid that engaged in leaping and vertical clinging as part of its locomotor repertoire as suggested by its morphological similarities with the callitrichines. The obtained body mass estimate is 352 g, which is within previously proposed ranges (i.e., 250–500 g; Marivaux et al., 2012), and similar to some of the extant callitrichines.

The Río Cisnes talus from the Chilean site of Alto Río Cisnes is currently taxonomically unassigned and dates to the Friasian South American Land Mammal Ages (SALMA) ∼16 Ma (Tejedor, 2003). This talus is about the size of that of *Pithecia*, and has been described as being morphologically similar to that of *Callicebus* or a smaller version of a *Carlocebus* talus (Tejedor, 2003, Tejedor, 2008). The analyses performed here suggest that the Río Cisnes talus shows a similar morphology to that observed in *Aotus*, *Proteropithecia* and *Neosaimiri*. The CVA classified this talus as similar to the Cebidae. It has been suggested that the moderately high talar body with the parallel-sided rims and the relatively long neck could be associated with increased leaping in what otherwise looks to be a generalized arboreal quadruped (Gebo and Simons, 1987, Meldrum, 1990). The locomotion CVA is in agreement with this proposal. Finally, the first body mass estimate of 1509 g for this fossil was provided, which is similar to other fossils and to the largest *Callicebus* species and the smallest *P. pithecia*.

*Proteropithecia neuquenensis*, a medium-sized platyrrhine known from a single talus and isolated teeth, was found in the Collón Curá formation in Neuquén, Argentina and based on dental traits has been classified as a pitheciin ancestor (Kay et al., 1998). The *P. neuquenensis* talus has been described as exhibiting a general similarity to *Callicebus* or *Aotus* (Ford, 1988, Meldrum, 1990). The PCA showed that *P. neuquenensis* occupies a position between the Patagonian and La Venta fossils, suggesting a potentially good representative for primitive talar morphology in some crown fossil taxa. The cluster analysis located it in a group with *Aotus*, Río Cisnes, *Proteropithecia* and *Callimico.* The CVA classified *P. neuquenensis* as belonging to Cebidae, however it also has a posterior probability of 0.278 of being classified as Pitheciidae. The talus has been described as having an oval head, moderate neck length, a wedged trochlea and an extended anterior proximal calcaneal facet, all of which have been interpreted as associated with the required ankle stability to perform arboreal quadrupedal activities and moderate leaping (Kay et al., 1998). That *P. neuquenensis* was classified as an arboreal quadrupedalist in the present study is consistent with these interpretations. The body mass prediction for this fossil was 2006 g, which is similar to some *Pithecia* species.

*Cebupithecia sarmientoi* is well represented in La Venta, Colombia. *Cebupithecia* was a medium-sized monkey with associated cranial, mandibular, and dental remains along with a partial skeleton; together the relatively complete *Cebupithecia* fossils suggest a phylogenetic position within Pitheciinae (Hartwig and Meldrum, 2002). However, *Cebupithecia* lacks many Pitheciinae apomorphic postcranial characters (Fleagle and Meldrum, 1988, Ford, 1990, Hartwig and Meldrum, 2002). The PCA showed that *C. sarmientoi* is located on the morphospace near most owl monkeys, exhibiting a morphology similar to *Aotus nancymaae*. As was the case for *Proteropithecia*, the CVA classified *Cebupithecia* within Cebidae. The clustering analysis located it next to Madre de Dios, which is intriguing. *Cebupithecia* has been traditionally reconstructed as exhibiting mainly quadrupedal behaviors with moderate amounts of leaping, in a similar fashion to the cebines and *Callicebus* (Meldrum and Lemelin, 1991). Consistently, the CVA analysis using locomotor categories classified *C. sarmientoi* as an arboreal quadruped. The obtained body mass prediction is 1825 g, which is similar to previous predictions (i.e., 1602 g; Cooke et al., 2011) and to *P. pithecia.*

The analyzed specimen of *A. dindensis* was discovered within the Monkey Unit in the site of La Venta, Colombia (Setoguchi and Rosenberger, 1987, Gebo et al., 1990), and it was classified as a member of *Aotus,* due to its particular morphological characteristics, although it differs from the extant members of this genus in being smaller and having a slightly more square-shaped talar body (Gebo et al., 1990). This specimen exhibits a robust talar body, with parallel trochlear rims and only a slight proximal wedging (Gebo et al., 1990). Its trochlear surface is relatively flat, while the talar head and neck are very wide (Gebo et al., 1990). This combination of morphological features has been interpreted as being associated with an extensive use of arboreal quadrupedalism (Gebo, 1988, Gebo, 1989), with no indication of frequent climbing or leaping (Gebo et al., 1990). It is debated whether *A. dindensis* is an actual species or if it is conspecific with *Monhanamico hershkovitzi* (for further details see Kay, 1990, Rosenberger et al., 1990). Nonetheless, in the present study we subscribe to the classification of Gebo et al. (1990). *Aotus dindensis* is located near *N. fieldsi* in the morphospace, occupying a position within the locomotor ‘generalist’ area. The cluster analysis located this fossil within a group with most *Aotus*, Río Cisnes, *Proteropithecia* and *Neosaimiri*. In the family CVA, this specimen was classified as a member of the Pitheciidae, while the locomotor analysis categorized it as an arboreal quadrupedal species, as previously suggested by Gebo et al. (1990). The average body mass prediction for *A. dindensis* is 873 g, thus being only slightly smaller than previous predictions (i.e., 1000 g; Cooke et al., 2011).

A number of postcranial specimens belonging to *N. fieldsi* have been discovered at La Venta, Colombia, and interpreted as ancestral to the extant genus *Saimiri* (Stirton, 1951, Szalay and Delson, 1979, Rosenberger et al., 1990, Takai, 1994). The talar morphology of *Neosaimiri* has been described as exhibiting parallel trochlear lips, a narrow trochlear surface, a relatively small and flattened talar head and moderately long talar neck (Nakatsukasa et al., 1997). Similarities in postcranial morphology between *Neosaimiri* and *Saimiri* suggest arboreal quadrupedalism to be its predominant locomotor behavior, although it probably engaged in leaping with relative frequency (Gebo et al., 1990, Meldrum et al., 1990). The PCA showed that *Neosamiri* is similar to some *Cebus* species, *Cacajao* and *A. dindensis* based on the two first PC axes. The family CVA classified *Neosaimiri* as Cebidae, while its inferred main locomotor behavior was arboreal quadrupedalism. The average body mass predictions for multiple individuals range between 758 and 871 g, which is only slightly larger than published dental predictions (i.e., 725 g; Cooke et al., 2011).

*Paralouatta marianae* was designated on the basis of a single talus from the Early Miocene locality of Domo de Zaza, Cuba (MacPhee et al., 2003). This talus has been described as being only subtly different from that of *Paralouatta varonai* even though 17–18 Ma allegedly separate them (MacPhee and Meldrum, 2006) and *P. marianae* is significantly smaller. There is no good morphological comparison for the talus of *Paralouatta* among extant NWM (MacPhee and Iturralde-Vinent, 1995). MacPhee and Iturralde-Vinent (1995) particularly noted that the Atelidae differ from *Paralouatta* in having a ‘wedged’ trochlea with a low trochlear relief, which would be related to maximizing mobility at the talocrural joint, whilst *Paralouatta* exhibits a talus more suited for stability rather than mobility. The talus of *Paralouatta* has a clearly noticeable cotylar fossa facing an extended medial malleolus articular surface, thus offering a stable seating for the medial malleolus (MacPhee and Iturralde-Vinent, 1995). The cotylar fossa, which is typically absent in large-bodied platyrrhines, is present in Old World monkeys such as *Theropithecus*, hence the suggestion of semiterrestriality in *Paralouatta* (MacPhee and Meldrum, 2006). The PCA showed that *Paralouatta* occupied a position close to *Alouatta*, as well as to some of the oldest Patagonian fossils (i.e., *Soriacebus, Dolichocebus* and *Carlocebus*). The hierarchical clustering analysis located this fossil close to *Cebus* and *Dolichocebus, Carlocebus* and *Soriacebus*. The family CVA classified *Paraloutta* within the Cebidae, while the locomotion CVA categorized it as an arboreal quadruped. In terms of locomotion, the results suggest arboreal quadrupedalism, however the analyses lacked terrestrial or semiterrestrial categories so it is not possible to rule out these potential specializations. Further analyses considering terrestrial Old World monkeys would be required to test this possibility. The body mass prediction carried out in this study for *P. marianae* employed highly accurate postcranial surface area regressions to compute the first body mass data for this specimen, which predicts 4708 g for this taxon. This value is similar to previous body mass predictions for *Antillothrix bernensis* based on craniodental measurements (i.e., 4.7 kg; Rosenberger et al., 2011), thus being slightly smaller than the extant Alouattinae species.

### 4.3 Locomotor mode percentages

The PLS analyses provide strong evidence for the association between talar shape and locomotion (measured as LMP); therefore talar shape can be used to infer locomotion. The talus is primarily stiffened by trabecular networks (unlike the diaphysis in long bones) and is the principal mechanical connection between the leg and the foot (Parr et al., 2013); it not only transmits the forces derived from the body mass, but also provides stability and/or mobility for the hind limbs during diverse postural and locomotor behaviors (Boyer et al., 2015). Many authors have proposed that mechanical loading regulates trabecular remodeling (e.g., Carter et al., 1987, Turner, 1998, Zadpoor et al., 2012), therefore different locomotor repertoires would have exerted differential loading regimes on the talus, thus gradually shaping it during NWM evolution.

In terms of locomotion reconstruction, all of the present analyses are consistent with the suggestion that the ancestral condition for the platyrrhines was predominantly arboreal quadrupedal. The PCA of the LMPs (Fig. 7a) showed that there is a good separation of groups. The groups cluster according to locomotor categories, principally distinguishing between the more specialized or derived forms along the respective axes. Large-bodied taxa using climbing/suspension (i.e., atelids) were distinguished from small-bodied species using claw-climbing, clinging and vertical leaping (i.e., callitrichines) along PC1, while PC2 separated between medium-sized NWM characterized by different levels of quadrupedalism, with some taxa occupying a central more ‘generalist’ position. The mapping of the PC1 of the LMPs on the platyrrhine phylogeny showed that the ancestral condition exhibited values similar to those expected for predominantly quadrupedal taxa, and that both the suspensory/clamber and leaper/vertical clinging locomotor repertoires evolved posteriorly in two different groups of NWM (i.e., atelids and callitrichines, respectively). The same procedure was repeated for PC2, which showed a distinction between the less quadrupedal genera (e.g., *Ateles*, *Callithrix, Callimico*), and those that exhibited higher levels of quadrupedalism. Interestingly, *Saimiri* and *Callicebus* showed the highest level of quadrupedalism (i.e., lowest PC2 score), thus repeating the convergence scenario found by the SURFACE method. For this variable, the ancestral state reconstruction was also found to be a quadrupedal condition, although not as specialized as in *Saimiri* or *Callicebus*, but more ‘generalist’ such as the Pitheciinae *Chiropotes* and *Cacajao*, the Callitrichinae *Saguinus* and *Leontopithecus* or even *Alouatt*a.

### 4.4 Evolutionary modeling

The present model selection results show that it is possible to explain talar shape diversification by invoking an OU model of adaptive peak shifts to three optima, defined by the different platyrrhine families. The OU-Clade model — a fully phylogenetic hypothesis where each platyrrhine family occupied a separate adaptive peak — was the best supported among all the tested hypotheses. This is consistent with the structuring of the data in the shape phylomorphospace (Fig. 9) where the platyrrhine families occupy mainly three distinct areas. This result means that each platyrrhine family has its own talar shape optimum, which could be associated with the previously described locomotor categories (climbing/suspension in Atelidae, arboreal quadrupedalism in Pitheciidae, and leaping in Cebidae), but also to other ecological differences such as canopy levels or diet. Nonetheless, some members of the Cebidae are more quadrupedal; hence this result is intriguing. One possibility is that *Cebus*, *Saimiri* and *Aotus* exhibit an ancestral talar morphology on its way towards the optimum nearer the callitrichines, or simply that the first five PCs do not totally represent the subtleties of shape variation in the platyrrhine family. In any case, the obtained results in combination with the DTT plot suggest that talar morphological diversification gradually differentiated into three distinct areas of the morphospace that are related mainly to phylogenetic clades (with some slight convergence between *Callicebus* and *Saimiri* as observed in the phylomorphospace and the SURFACE model). Some species seem to retain the primitive morphology similar to the one observed across the fossil sample (e.g., *Cebus* and *Cacajao*). In contrast, the least supported model was the OU1 model, suggesting that there is not a single unique adaptive optimum for talar shape in the NWM.

Talar centroid size followed the pattern observed in previous research regarding platyrrhine body mass (Aristide et al., 2015) and brain shape (Aristide et al., 2016), where there were several unique and shared optima, mainly defined by the multidimensional ecological niche hypothesis (i.e., OU-Multidimensional niche), which combined both diet and locomotion information (Rosenberger, 1992). As found by these previous studies (Aristide et al., 2015, Aristide et al., 2016), it seems that talar centroid size – a generally good proxy for body mass (Halenar, 2011) – evolved in the platyrrhine radiation initially by a rapid diversification, as observed in the DTT plot of centroid size. This is similar to the trend observed for body mass by Aristide et al. (2015), likely because both are scale measurements that are highly correlated. This relationship was likely associated with a differentiation among NWM families within an ecological adaptive landscape mostly defined by locomotion and diet (Rosenberger, 1992, Aristide et al., 2015). It has been previously proposed that size diversification in platyrrhines was mostly related to diet variation (Marroig and Cheverud, 2001, Perez et al., 2011), however the present results align with other findings that support a more complex scenario where platyrrhine evolution among the main lineages is linked to size changes related to a multidimensional niche (Rosenberger, 1980, Rosenberger, 1992, Aristide et al., 2015, Aristide et al., 2016). Nonetheless, it is important to note that even though the diet ecological dimension alone is not enough to explain platyrrhine centroid size and body mass diversification, the other best supported models for centroid size is related to diet (i.e., OU-Diet Composition). The locomotion model alone was poorly supported. Perhaps this indicates the relative contribution of these different factors to the OU-Multiple Niche model, although further investigations are required. The DTT plot shows how centroid size disparity is high during the early branching of the phylogeny, possibly related to changes in ecological opportunity (Harmon et al., 2003). The magnitude of the centroid size disparity is strikingly high during the early branching processes (Figure 8, Figure 10b), similar to that found by Aristide et al. (2015) for body mass, thus supporting again the distinctiveness of the platyrrhine radiation (Delson and Rosenberger, 1984). Interestingly it seems that this early differentiation in size was not coupled with immediate changes in talar shape, but that these structural changes occurred gradually following the different NWM family differentiations. The fossil evidence supports these results since the different morphological analyses showed that most fossils exhibit a generalist and possibly primitive morphology, while showing significant size variation according to the obtained predictions ranging from 352 g (Madre de Dios) to 4708 g (*P. marianae*). This is consistent with previous results that have suggested that body size partitioning in platyrrhines is already evident in ancient lineages (Aristide et al., 2015).

One of the main predictions of an adaptive radiation hypothesis is that phenotypes diversify early in the branching process of the phylogeny in relation to certain ecological factors (Schluter, 2000, Losos, 2011). Previous eco-functional studies have indicated that there are natural size thresholds structuring platyrrhine locomotor-dietary niches (Rosenberger, 1992, Youlatos and Meldrum, 2011, Fleagle, 2013). The ecological opportunity that existed during the early evolutionary history of platyrrhines was most likely a significant factor influencing body size changes among the main clades as observed in both the centroid size and body mass traitgrams and DTT plots (Figs. 8 and 10) (Aristide et al., 2015). The present results support that along with this initial diversification in body size, likely due to ecological opportunity, there was probably a subsequent gradual differentiation in talar shape (as observed in Figs. 9 and 10a). These shape changes in talar morphology were more marked in the two lineages that evolved notably different locomotion repertories compared to the ancestral condition (i.e., atelids and callitrichines), while other groups still exhibit a talar shape relatively similar to the one observed in most of the analyzed fossils (e.g., *Chiropotes*, *Cacajao*, *Cebus*).

### 4.5 Implications for platyrrhine evolution

The placement of the fossil species on the PCA (Fig. 4) showed that most extinct taxa occupy the central area defined by quadrupedal ‘generalist’ species (an area occupied by some extant species exhibiting different frequencies of additional climbing or leaping behavior). This is consistent with the CVA and the ancestral trait reconstruction for the LMPs that indicated that the ancestral platyrrhine condition was probably predominantly quadrupedal with only minor contributions from other more specialized locomotor behaviors. Nonetheless, until the recovery of postcranial elements for the earliest platyrrhine fossils (e.g., *Branisella* and *Perupithecus*), not much can be said with certainty about the ancestral locomotor condition of the very first platyrrhines, especially if these fossils are considered to belong to an ancient radiation of stem platyrrhines that did not lead to crown NWM (Rosenberger et al., 1991, Takai et al., 2000, Kay et al., 2008). This would imply that studying the locomotor diversity observed in the extant NWM would point to the ancestral condition of the last common ancestor of modern platyrrhine species, rather than the earliest ancestor of all platyrrhines (i.e., extinct and extant) (Ford, 1988, Youlatos and Meldrum, 2011).

Due to the absence of post-cranial material belonging to the oldest found platyrrhines, it is perhaps relevant to discuss the obtained results in relation to other primate fossils that have known tali. Platyrrhines are considered to be a monophyletic group that emerged during the African Eocene (Ciochon and Chiarelli, 1980, Houle, 1999, Oliveira et al., 2009), and most of the primate fossil evidence for that time period comes from three groups from the Fayum of Egypt (i.e., the propliopithecids, the oligopithecids and the parapithecoids) (Fleagle and Simons, 1982, Fleagle and Simons, 1983, Seiffert et al., 2000, Simons, 2004). Among these fossils, it has been proposed that *Apidium* (Hoffstetter, 1980, Ford, 1990, Fleagle and Kay, 1994, Takai et al., 2000) or *Proteopithecus* (Simons, 1989, Simons, 1997, Simons and Seiffert, 1999, Gladman et al., 2013) might represent the ancestral NWM morphotype better. *Apidium* is usually interpreted as being a frequent leaper (Fleagle and Simons, 1983, Fleagle and Simons, 1995, Gebo et al., 2000, Gebo et al., 2012; although for a different opinion see Ryan et al., 2012), while *Proteopithecus* has been described as relying on agile quadrupedal locomotion, probably also involving some pronograde leaping (Gebo et al., 1994, Simons and Seiffert, 1999, Seiffert et al., 2000, Ryan et al., 2012), therefore it might be speculated that the ancestral platyrrhine was a leaper. Nonetheless, the shape of the oldest Miocene talus analyzed here (i.e., *Dolichocebus*) has been described as distinctively different from the Fayum fossils (Gebo and Simons, 1987) and the present results indicate that all the oldest materials are more similar to the ‘generalized’ shape of *Cebus* rather than to specialized leapers such as the Callitrichinae (Figs. 4 and 6). In addition, leaping behavior is notoriously associated with size. Thus, the smaller the body size of the ancestral platyrrhine, the more likely leaping may be a factor. From the traitgrams in Figure 8 it is notable that the ancestral centroid size and body mass reconstruction for the ancestral platyrrhine condition (i.e., root of the phylogeny) corresponds to the body mass of *Cebus* (∼3000 g), while its talar size is similar to *Pithecia monachus*. However, this analysis estimates the ancestral size condition using the data from only the modern NWM, which represent only a subset of all Platyrrhini through time. Furthermore, the ancestral state reconstructions have the known limitation that the probability of computing the correct ancestral condition decreases as the temporal depth increases (Martins and Cunningham, 1999). Therefore caution is required when extrapolating this result. Furthermore, when reconstructing locomotor behaviors, it is mostly the dominant locomotor modes that are reconstructed and not the entire repertoire of positional behaviors (MacPhee and Meldrum, 2006). For instance, saying that the ancestral locomotor condition of the platyrrhines was most likely arboreal quadrupedalism does not imply that this specimen was incapable of a wide variety of behaviors (such as leaping, climbing, running, suspension, and clambering), but rather that arboreal quadrupedalism was its predominant locomotor mode (MacPhee and Meldrum, 2006). In summary, the present results point to an ancestral morphological pattern that can be described as a generalized, medium-sized, arboreal quadruped as has been previously suggested (Ford, 1988, Gebo et al., 1990, Tallman and Cooke, 2016).

Even though the present research did not attempt to resolve the debate regarding the LLH and SPH, the results do provide some interesting insights to trigger further research. The early Miocene fossils analyzed here from Patagonia have been hypothesized to represent either a distinct ancient radiation or the early ancestors of the modern clades (Rosenberger et al., 2009). The results show that all these fossils (i.e., *Dolichocebus, Soriacebus* and *Carlocebus*) clustered together along with *Paralouatta and* some generalized species (i.e., *Cebus*) (Fig. 6). This can be interpreted according to the two existing competing hypotheses in the following manner. Under the SPH perspective, both the basal fossil platyrrhines and the ancestors of the living NWM would have exhibited a primitive morphology associated with a more ‘generalist’ arboreal quadrupedalist locomotor behavior. This implies that the fossil forms were adapted to niches in the early Miocene southern forests analogous to those of the ancestral forms of the extant NWM (i.e., a convergence scenario). Another possible interpretation under the SPH perspective is that rather than convergent evolution, the observed morphological pattern could just be the retention of characteristics from an older ancestor. Therefore, even if there was a stem radiation followed by the modern crown radiation, the modern radiation had to come from one of the stem taxa, thus the observed similarity in talar morphology could be merely the retention of ancestral traits. On the other hand, under the LLH, the fact that most fossils exhibit a primitive morphology is explained by noting that these fossils might represent the ancestral forms leading to the extant lineages or members of the same long-lived lineages. It is important to bear in mind that the present study focused on only one anatomical structure, the talus, hence these results are limited and caution is required when extrapolating these results to reconstruct the evolutionary history of platyrrhines.

## 5 Conclusion

In spite of the numerous studies and decades of research, a comprehensive understanding of the evolutionary history of platyrrhines is still lacking. This is highlighted by the continued debates on the proto-platyrrhine immigration to South America (Houle, 1999, Oliveira et al., 2009, Cachel, 2015), on the issue regarding the SPH and LLH hypotheses (Kay et al., 2008, Kay and Fleagle, 2010, Rosenberger, 2010, Perez and Rosenberger, 2014, Kay, 2015b) and on the phylogenetic position of the genus *Aotus* (Menezes et al., 2010, Rosenberger and Tejedor, 2013, Aristide et al., 2015). Whilst this study does not provide definitive answers to any of these major questions, it does provide additional context. In particular it shows that locomotor behavior has a strong influence on talus morphology and it indicates that the earliest NWM had a generalized quadrupedal lifestyle as has been previously proposed (e.g., Ford, 1988, Tallman and Cooke, 2016) and that the ancestral platyrrhine was probably medium-sized (reconstructed body mass: 2966 g; 95% LCI: 1623 g; UCI: 4309 g). Platyrrhines subsequently seemed to evolve towards three different selective optima, represented by the three main locomotion habits observed in extant NWM. In addition, new body mass predictions for all the analyzed Miocene platyrrhines were provided, which show that during the Miocene there was already a noticeable size variation. The present work represents a contribution to the understanding of platyrrhine evolution by applying a combination of GM and comparative techniques in order to understand the evolution of one of the best-represented structures in the platyrrhine fossil record, the talus. This allowed not only to reconstruct aspects of the locomotor behavior of fossil individuals, but also provided information about the evolution of the locomotor diversity observed in extant platyrrhines, its relationship with talar size and shape, and its relation with the adaptive radiation that platyrrhines experienced.
