# Supplementary material for: The evolution of the platyrrhine talus: A comparative analysis of the phenetic affinities of the Miocene platyrrhines with their modern relatives
Source: J Hum Evol. 2017 Oct;111:179–201. doi: 10.1016/j.jhevol.2017.07.015 (PMC5603972; doi:10.1016/j.jhevol.2017.07.015)
Supplement: Supplementary file 1 [file mmc1.pdf]

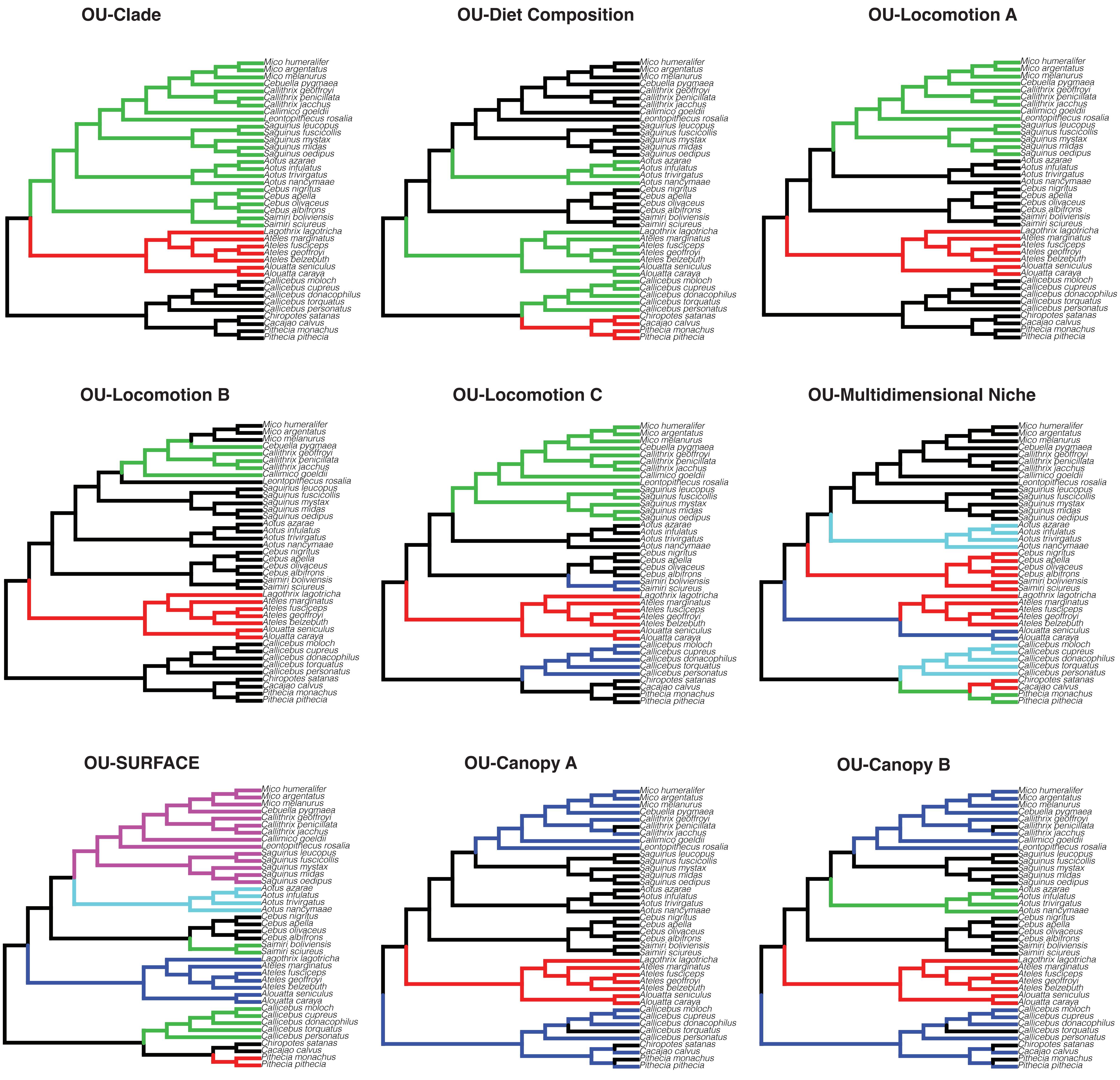

S4. Alternative multivariate OU hypotheses for the evolution of platyrrhine talar shape and size. The phylogenetic trees represent the different multi-regime OU hypotheses included in the model selection analyses carried out in 'mvMORPH'. Additionally to these multi-regime OU models, a single-peak OU (OU1), Brownian motion (BM), and early-burst (EB) models were also fitted.

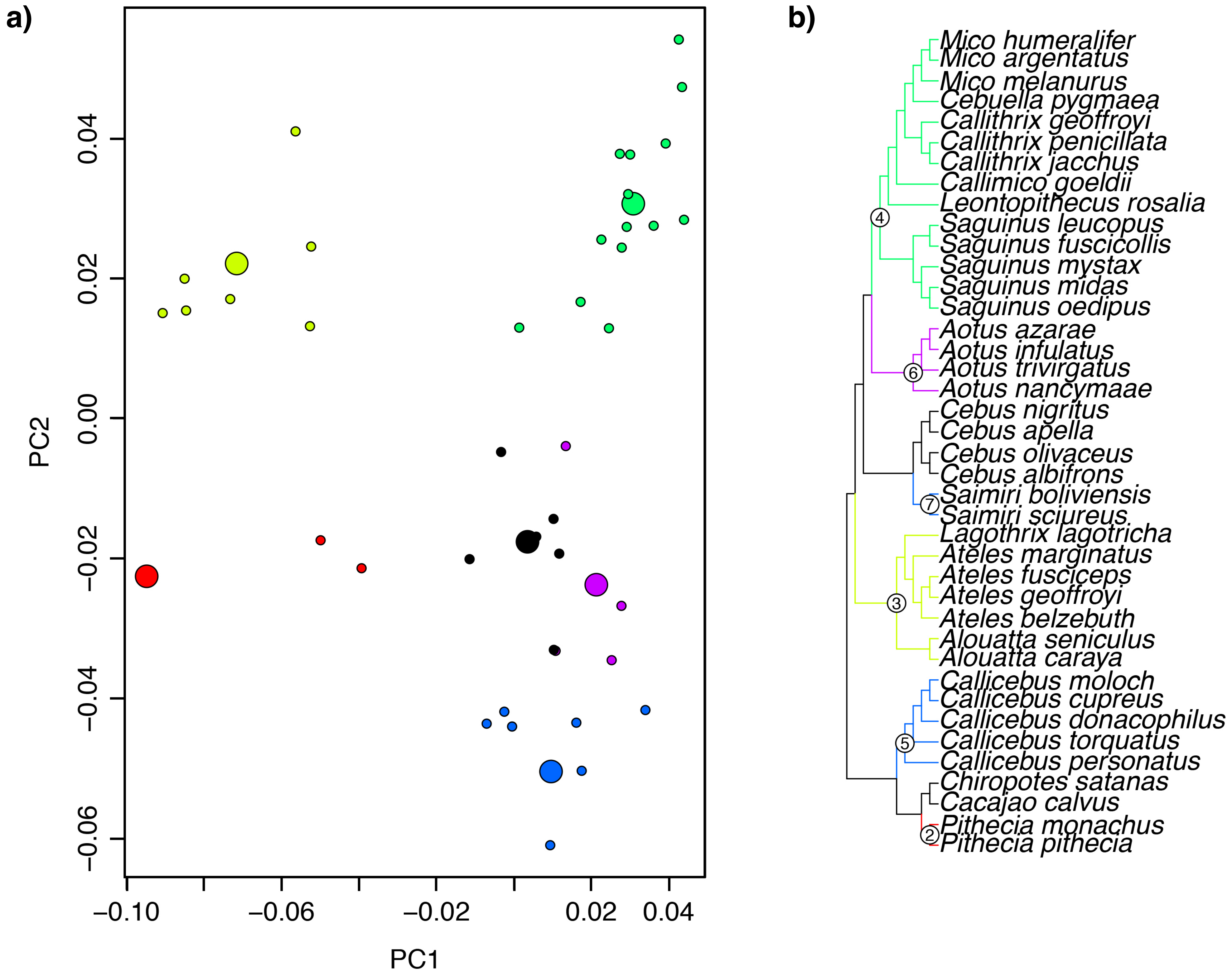

S7. Results of the SURFACE method: Adaptive regimes for the best-fitting model found by SURFACE are mapped on the a) morphospace defined by PC1 and PC2 and on the b) phylogeny. Large circles in a) represent the position of the estimated optima for each regime, while the small dots are the species values in the morphospace. Numbers at nodes in b) represent the different regimes found by the SURFACE method.
